# The frictional layer in the observed momentum budget of the trades

**DOI:** 10.1002/qj.4364

**Published:** 2022-10-01

**Authors:** L. Nuijens, A. Savazzi, G. de Boer, P‐E. Brilouet, G. George, M. Lothon, D. Zhang

**Affiliations:** ^1^ Geoscience and Remote Sensing Department Delft University of Technology Delft the Netherlands; ^2^ Cooperative Institute for Research in Environmental Sciences University of Colorado Boulder Boulder Colorado; ^3^ Physical Sciences Laboratory NOAA Boulder Colorado; ^4^ Integrated Remote and In Situ Sensing University of Colorado Boulder Boulder Colorado; ^5^ CNRM University of Toulouse, Météo‐France, CNRS Toulouse France; ^6^ Max‐Planck Institute for Meteorology Hamburg Germany; ^7^ Laboratoire d'Aérologie University of Toulouse, CNRS, UPS Toulouse France; ^8^ CICOES University of Washington Seattle Washington; ^9^ Pacific Marine Environmental Laboratory NOAA Seattle Washington

**Keywords:** eddy momentum fluxes, momentum budget, trade wind convection

## Abstract

Profiles of eddy momentum flux divergence are calculated as the residual in the momentum budget constructed from airborne circular dropsonde arrays (∼220 km) for 13 days during the EUREC${}^{4}$A/ATOMIC field campaign. The observed dynamical forcing averaged over all flights agrees broadly with European Centre for Medium‐Range Weather Forecasts (ECMWF) Integrated Forecasting System (IFS) forecasts. In the direction of the flow, a mean flux divergence (friction) exists over a 1.5‐km deep Ekman layer, and a mean flux convergence (acceleration) is present near cloud tops. The friction is countergradient between 1 and 1.5 km, where vertical wind shear exceeds the observed thermal wind. From the frictional profile, a 10‐m momentum flux of ∼0.1 N·m${}^{-2}$ is derived, in line with Saildrone turbulence measurements. A momentum flux divergence in the cross‐wind direction is pronounced near the surface and acts to veer the wind, opposing the friction‐induced cross‐isobaric wind turning. Weaker friction and upper‐level acceleration of easterly flow are observed when stronger winds and more vigorous convection prevail. Turbulence measurements on board the SAFIRE ATR‐42 aircraft and the Uncrewed Aircraft System (UAS) RAAVEN reveal pronounced spatial variability of momentum fluxes, with a non‐negligible contribution of mesoscales (5–30 km). The findings highlight the nontrivial impact of turbulence, convection, and mesoscale flows in the presence of diverse cloud fields on the depth and strength of the frictional layer.

## Graphical abstract

The momentum transport by turbulence, convection, and mesoscale flows within a ∼200‐km area is analyzed from the observed momentum budget in the trades. The wind is decelerated over a layer ∼1.5 km deep, but friction‐induced ageostrophic wind turning is weak. An acceleration of wind is observed in the upper cloud layer and near the trade inversion. The observations suggest that convection and mesoscale circulations play a key role in the observed momentum flux divergence.

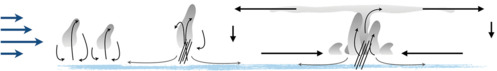

## 1 INTRODUCTION

Strong easterly winds near the surface prevail over much of the subtropical and tropical oceans. The trade winds are important because they define convergence patterns in the Tropics, where the ascending branch of the Hadley circulation produces the majority of tropical rainfall. The trade winds also modulate ocean currents and upwelling, sea‐surface temperatures, and turbulent fluxes at the ocean surface.

Surface wind speed correlates with trade‐wind cloud amount and precipitation, as well as with patterns of organization on synoptic (Klein, 1997; Brueck *et al*., 2014; Nuijens *et al*., 2015) and diurnal time‐scales (Vial *et al*., 2019; 2021), where it is typically considered an “external” large‐scale controlling factor. However, this overlooks the fact that turbulence, convection, and cloudiness also impact winds.

Turbulence and convection modify the wind profile directly through turbulent momentum transport, precipitation, and cold pools. The friction introduced by eddy momentum fluxes leads to ageostrophic, cross‐isobaric flow in the so‐called Ekman layer that helps define the Intertropical Convergence Zone (ITCZ). More indirectly, the transport of heat and moisture by turbulence and convection, and cloudiness and radiative cooling, help set the thermal contrast between the subtropics and Tropics and thus the large‐scale pressure gradients that drive the trade winds (Riehl and Malkus, 1957). In the absence of a strong Coriolis force in the inner Tropics, momentum transport may also diffuse gravity waves that act to smooth horizontal temperature gradients (Kuang, 2012; Nuijens and Emanuel, 2018).

Eddy momentum fluxes are not straightforward to measure from the smallest turbulent scales to mesoscale circulations associated with convection, especially not at height levels beyond meteorological towers and over remote oceans. As large areas of the (sub)tropical atmosphere remain void of wind and momentum‐flux measurements, the profile of eddy momentum flux divergence and its role in the trade‐wind momentum budget has not been frequently studied. Inspired by a wealth of observations collected during the EUREC^4^A/ATOMIC field campaign (Stevens *et al*., 2021), the objective of this study is to revisit the trade‐wind momentum budget. In particular, we are interested in whether trade‐wind convection produces significant “cumulus friction” beyond the turbulent mixed layer (a term first introduced by Schneider and Lindzen (1976) to denote the effect of convective momentum transport (CMT) from deep convection). In other words, we investigate variations in the depth and magnitude of the frictional layer.

Much of what we know about the trade‐wind momentum budget stems from shipborne sounding arrays in the 1970s. During the Atlantic Trade Wind Experiment (ATEX) field campaign, three ships drifted 750 km apart in a triangle constellation, and radar tracking of three‐hourly radiosonde balloons was used to determine wind‐speed profiles, providing the first observational evidence of the existence of divergence (Brümmer *et al*., 1974). In their study, Brümmer *et al*. (1974) interpreted the residual in the observed momentum budget as the friction produced by turbulent eddies across all scales, and found it to extend well beyond the mixed layer. Using the assumption that (turbulent) stresses are zero at the wind maximum, they integrated the profile of friction to derive the total shearing stress τ at the surface. The value they obtained was much lower than what was measured using direct eddy covariance techniques on the ships. This led them to hypothesize that organized convective motions in the subcloud layer contribute considerably to the vertical flux of momentum through the mixed‐layer top and into the cloud layer, making their assumption of vanishing stress at the local wind maximum invalid. Using a similar method, Holland and Rasmusson (1973) derived the budget from about 15 soundings per day launched from four ships during the Barbados Oceanographic and Meteorological EXperiment (BOMEX), which led to an estimated frictional layer that spanned 60–76% of the trade‐wind layer (up to the trade inversion).

Carr and Bretherton (2001) used European Centre for Medium‐Range Weather Forecasts (ECMWF) and National Centers for Environmental Prediction (NCEP)–National Center for Atmospheric Research (NCAR) reanalyses to calculate CMT as a momentum budget residual over several tropical oceanic regions. They found a significant zonal momentum residual above the mixed layer, hinting at an important role for shallow convection. Similarly, Lin *et al*. (2008) found that, in the suppressed branch of the tropical Walker circulation, CMT must play an important role to balance pressure gradients in the absence of a large Coriolis force and without a large role for advection. Other studies have used the conceptual mixed‐layer model framework to show that a flux of momentum through the mixed‐layer top (cloud base) is necessary to explain the observed surface wind climatology in the Tropics (Deser, 1993; Chiang and Zebiak, 2000; Stevens *et al*., 2002).

A handful of studies have used large‐eddy simulations (LESs) to study momentum transport in the trades (Schlemmer *et al*., 2017; Larson *et al*., 2019; Dixit *et al*., 2020; Helfer *et al*., 2020), in cold‐air outbreaks (Saggiorato *et al*., 2020), and in well‐known case studies of both shallow and deep convection (Zhu, 2015). The latter two studies also decomposed momentum flux profiles by wave number (eddy size) using fast Fourier transforms. Although the precise contribution of different eddy scales to the momentum flux depends strongly on the horizontal grid size and the subgrid turbulence closure, these studies suggest that in shallow cumulus regimes shear‐driven turbulent eddies (with scales less than ∼200 m) dominate in the surface layer, where they act to slow down the flow, while larger eddies (with scales ∼500 m and larger) carry almost all of the flux above the surface layer and in the mixed layer up to cloud base. These larger eddies act to accelerate the wind in the lower half of the mixed layer. In the cloud layer, both small and large eddies carry a significant portion of the momentum flux, but sometimes with a different sign. While small eddies are diffusive of nature with so‐called downgradient transport, larger eddies carry momentum in the opposite countergradient direction. A layer of countergradient transport is notably deeper and more pronounced in nested LES hindcasts (Dixit *et al*., 2020; Helfer *et al*., 2020) than in traditional LESs with cyclic boundary conditions (Schlemmer *et al*., 2017; Larson *et al*., 2019), which is attributed to eddy momentum fluxes generated by horizontal circulations on mesoscales (20–200 km) that are inhibited in cyclic LES domains. LESs of deep convection have also shown considerable sensitivity of CMT to the size of the simulation domain and its lateral boundary conditions, which determine the mesoscale pressure gradients that can develop (Badlan *et al*., 2017). It is unclear whether the LESs used so far to develop CMT parameterizations for global models capture all the flows relevant for CMT in nature, which motivates a study of the influence of CMT on wind using observations as a starting point.

The central element circular dropsonde arrays performed during EUREC${}^{4}$A/ATOMIC were designed specifically to obtain confident estimates of mesoscale divergence, pressure, temperature, and humidity gradients over an area ∼222 km in diameter (Bony and Stevens, 2019; George *et al*., 2021b), as required to construct the heat, moisture, and momentum budgets following the seminal ATEX and BOMEX studies. The pressure sensors and GPS receivers carried by modern dropsondes reduce measurement uncertainties, particularly in measuring the pressure gradient, which plagued early budget studies. In the context of these observations, we interpret the mean wind as that averaged over the EUREC${}^{4}$A/ATOMIC circle, which is driven by the pressure gradient, Coriolis force, and advection determined over the circle. All wind fluctuations on smaller scales, produced by turbulence, CMT, and gravity waves, are assumed to contribute to the budget residual, which is interpreted as an eddy momentum flux divergence. To validate the inferred eddy momentum flux profiles, we make use of in situ turbulence measurements collected in the mixed layer and lower cloud layer by the ATR‐42 (hereafter referred to as ATR), operated by the French Service des Avions Français Instrumentès pour la Recherche en Environnment (SAFIRE) (Bony *et al*., 2021) and the Uncrewed Aircraft System (UAS) CU RAAVEN operated by the University of Colorado (de Boer *et al*., 2022), as well as surface momentum fluxes collected by a Saildrone, a wind and solar powered uncrewed surface vehicle (USV: Zhang *et al*., 2019).

The article is organized as follows: Section 2 describes the data sources, followed by a description and analysis of the vertical wind profile, circulation features, and wind diurnality in Section 3. The mean horizontal momentum budget is calculated and compared with the ECMWF Integrated Forecast System (IFS) in Section 4. In Section 5, we derive profiles of eddy momentum flux from the inferred frictional force and compare these against the in situ measurements, followed by a discussion (Section 6) and conclusion (Section 7). Supporting Information on the prevailing circulation from ERA5 reanalysis has been provided with the online article.

## 2 EUREC${}^{4}$A/ATOMIC DATA

### 2.1 JOANNE dropsondes

**FIGURE 1:**
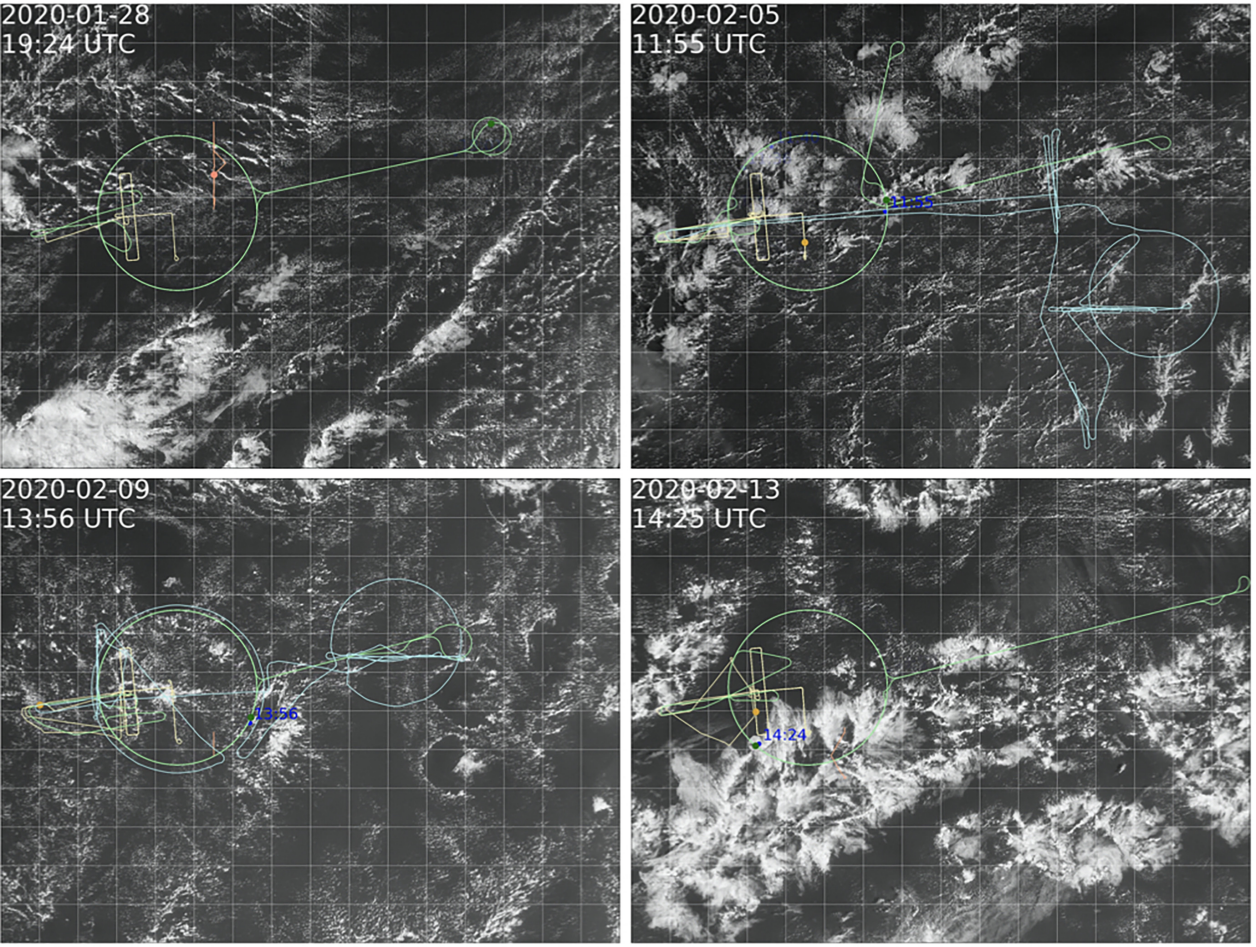
Snapshots of *GOES* visible satellite imagery at the time of HALO and ATR flight operations on January 28 and February 5, 9, and 13. Various platform tracks are overlaid in green (HALO), light blue (P3), yellow (ATR), and orange (RV Meteor). Times in blue indicate launched dropsondes along the HALO circle [Colour figure can be viewed at wileyonlinelibrary.com]

We use the EUREC${}^{4}$A/ATOMIC dropsonde dataset, named Joint dropsonde Observations of the Atmosphere in tropical North atlaNtic mesoscale Environments (JOANNE), which provides circle products as part of its Level‐4 data. All 85 circles were flown with dropsonde launches, out of which 70 circles flown by the German *High Altitude and Long Range* aircraft (HALO) were at a fixed location—the mean center at 57.67${}^{\circ }$W, 13.31${}^{\circ }$N and with a diameter of 222.82 km. We primarily use the measurements of these fixed circles, called EUREC^4^A‐circles after Stevens *et al*. (2021); see Figure 1. These circles were restricted to daytime measurements between 1000 and 2300 UTC. There were 13 flight days and a typical flight included flying two sets of three circles each, with an excursion of around 1 hr in between the two sets. This strategy allows for the sampling of the same region over a period of 7–8, hr, therefore providing an Eulerian perspective of the airmasses moving through the region.

We also explored dropsonde measurements from the 15 circles flown by the P3 aircraft, which provide the advantage of night‐time sampling, but, as the P3 flew only two subsequent circles each day, important information about time evolution is missing. More details about the circles flown by HALO and P3 are provided by Konow *et al*. (2021) and Pincus *et al*. (2021), respectively, whereas the description of the circle products from the JOANNE dataset is found in George *et al*. (2021b).

The circle products include area‐averaged quantities of parameter gradients, divergence, and vertical velocity. The gradients are estimated by the regression method as described in Bony and Stevens (2019), after Davies‐Jones (1993), Lenschow *et al*. (2007), and Helms and Hart (2013). For any parameter φ measured from the circle dropsondes, the area‐averaged gradients in the zonal ($\partial_{x}\varphi$) and meridional ($\partial_{y}\varphi$) are estimated as

(1) $$\partial_{x}\varphi \Delta x_{i}+\partial_{y}\varphi \Delta y_{i}=\varphi_{i}-\varphi_{o},$$

where $\Delta x_{i}$ and $\Delta y_{i}$ are the eastward and northward displacements of the ith dropsonde from the mean coordinates of all dropsondes in the circle, that is, effectively the center of the circle, $\varphi_{i}$ is the local value measured by the dropsonde, and $\varphi_{o}$ is the area mean. Furthermore, JOANNE provides uncertainty at a given altitude for gradient terms ($\partial_{x}\varphi$) by estimating the residual standard error for the linear regression used to compute the gradients, which can be defined as

(2) $$\mathrm{Residual}\;\mathrm{standard}\;\mathrm{error}=\frac{\sqrt{\sum_{i=1}^{n}{\left(\varphi_{i,\text{obs}}-\varphi_{i,\text{est}}\right)}^{2}}}{n-3},$$

where $\varphi_{i,\text{obs}}$ is the value measured by dropsonde i and $\varphi_{i,\text{est}}$ is the value computed for dropsonde i by using the gradients computed by linear regression provided in Equation 1. The n−3 in the denominator indicates the three degrees of freedom in Equation 1.

Solving Equation 1 for a system of all dropsonde measurements along the circle, the gradients $\partial_{x}\varphi$ and $\partial_{y}\varphi$ can be estimated with a least‐squares fit. The horizontal mass divergence (𝒟) is derived as

(3) $$𝒟=\partial_{x}u+\partial_{y}v,$$

where u and v are the zonal and meridional components of the horizontal wind.

The primary assumption behind this method is linearity in horizontal space and steadiness in time. Thus, the estimated gradients neglect small‐scale spatial variability. The steadiness in time is qualified given that the sampling time‐scale is short compared with the advection time‐scale (the aircraft flies fast, ∼190 m·s${}^{-1}$, compared with the speed of wind). Bony and Stevens (2019) showed this method to give almost identical results to the linear integral method that only assumes stationarity, at least for divergence.

### 2.2 Uncrewed aerial vehicle RAAVEN

In situ profiles of the subcloud layer are derived using measurements from the University of Colorado uncrewed aircraft system RAAVEN (de Boer *et al*., 2021). This 2.3‐m fixed‐wing platform was operated over the Atlantic Ocean from Morgan Lewis on the windward side of Barbados. The aircraft was operated in the near‐shore environment, generally flying around 1–2 km offshore, conducting regular profiling of the lowest 1,000 m between January 24 and February 15. The RAAVEN carries various sensors to measure the thermodynamic and kinematic states of the atmosphere, providing 10‐Hz temperature and wind measurements. With a slow air speed of ≈18 m·s${}^{-1}$, this corresponds to a sample spacing of ∼1.8 m. The platform was generally flown twice daily, with one flight taking place around 1000 local time (LT=UTC ‐ 4) and the second flight taking place around 1300 LT. For most flights, the same 2‐hr flight pattern was executed, including an initial profile from 20–1,000 m above mean sea level (MSL), followed by extended statistical sampling at a variety of altitudes, including 20‐min flight legs positioned just below the cloud‐base altitude and at 400, 200, and 20 m MSL, flying back and forth across distances of approximately 3–5 km. Additional details on the system and the dataset can be found in de Boer *et al*. (2022).

For the current study, observations from the extended (20‐min) statistical legs are used to derive eddy‐covariance‐based estimates of the turbulent momentum flux using the three‐component winds derived using the RAAVEN's onboard multihole pressure probe and inertial navigation system. To do so, winds are first rotated into a natural coordinate system for each leg by calculating the four‐quadrant inverse tangent of meridional (v) and zonal (u) winds:

(4) $$\begin{array}{ll} \theta_{s} & =\tan^{-1}({\bar{v}}_{\text{leg}},{ū}_{\text{leg}}), \end{array}$$

(5) $$\begin{array}{ll} \theta & =\tan^{-1}(v_{\text{leg}},u_{\text{leg}}), \end{array}$$

(6) $$\begin{array}{ll} \alpha_{T} & =\theta -\theta_{s}, \end{array}$$

where overbars represent mean quantities for that given leg. The angular offset $\alpha_{T}$ is then used to calculate the tangential wind at the altitude of a given leg:

(7) $$u_{s}=U\cos(\alpha_{T}),$$

where U is the wind speed. The momentum flux at this altitude is then calculated as

(8) $$\tau_{s}=-\rho \bar{u_{s}^{\prime }w^{\prime }},$$

where $w^{\prime }$ and $u_{s}^{\prime }$ are the detrended turbulent component of the vertical velocity and tangential wind, respectively, and ρ is the mean air density at a given level of flight.

### 2.3 SAFIRE ATR‐42

The HALO and SAFIRE ATR‐42 (ATR) flew a coordinated strategy (Figure 1), whereby the ATR flew within the circles at cloud base and in the subcloud layer to characterize the turbulence organization of the boundary layer (Bony *et al*., 2021). At the end of most flights, a short surface leg was performed at 60 m above sea level. The SAFIRE ATR‐42 was equipped with a five‐hole radome nose, as well as several temperature and moisture sensors, allowing for measurements of wind, temperature, and humidity at 25 Hz. For a true air speed of about 100 m·s${}^{-1}$, this corresponds to a sample spacing of approximately 4 m. Those turbulent fluctuations at 25 Hz are used here to compute the turbulent momentum fluxes over stabilized legs of 30 km, which is long enough to sample the structures that dominate the turbulent exchange and short enough to explore the spatial variability from one leg to another (Lenschow *et al*., 1994). Similarly to the RAAVEN, winds are first rotated into a natural coordinate system to obtain the along‐ and cross‐wind momentum fluxes (Equations (4), (5), (6), (7), (8)). For the period January 26–31, the vertical wind speed is not available and no fluxes are derived. More details on the ATR turbulence dataset can be found in Brilouet *et al*. (2021).

### 2.4 Saildrone

A NOAA funded Saildrone SD1064 was dedicated to the Trade Wind Alley between NTAS buoy and the HALO flight circle. The SD1064 continuously measured the winds at 5 m, air temperature and relative humidity at 2.3 m, and ocean currents between ∼−6 and −100 m, as well as the wave height and period, and downward solar and longwave radiation. Motion corrections of wind and ocean current measurements were done on board the Saildrone USV in real time (Zhang *et al*., 2019). Five‐minute averages of these measured state variables are used here to calculate the surface wind stress with the COARE3.6 bulk algorithm (Fairall *et al*., 2003; Edson *et al*., 2013). With the averaged Saildrone cruise speed of 2–3 kt, the 5‐min fluxes correspond to a spatial resolution of 500 m.

### 2.5 Ship‐borne wind lidar

A Leosphere long‐range Windcube (WLS70) was deployed on the RV Meteor during the entire campaign. It measured the line‐of‐sight radial velocity successively at four azimuthal positions along a cone angle of $14.7^{\circ }$ and at 20 height levels between 100 and 2,000 m, with one scan roughly every 30 s. The radial velocities were corrected for ship motions using an accompanying GPS system, described in Savazzi *et al*. (2021). After motion correction, the wind vector is retrieved and hourly averages are used to study the composite diurnal cycle.

### 2.6 IFS forecasts and ERA5 reanalysis

Operational high‐resolution (9 km) forecasts from the IFS as well as ERA5 reanalysis are used to assess the synoptic situation during EUREC${}^{4}$A/ATOMIC and evaluate the pressure gradient force and advection terms derived from the circular dropsonde arrays. For the forecast, model output was extracted at the nearest four neighbours of 61 points placed concentrically around the center of the EUREC${}^{4}$A/ATOMIC circle, matching the flight hours on flight days (Savazzi *et al*., 2021). ERA5 data are extracted for an area 65°–25°W and 0°–30°N encompassing Barbados. As described in Savazzi *et al*. (2021), the IFS and ERA5 are biased in their wind throughout the lower troposphere, with a maximum bias near the top of the trade‐wind layer and winds in the mixed layer that are too weak during the day and too strong during the night, a point we take into our discussion in Section 4.

## 3 THE MEAN WIND PROFILE AND VARIABILITY

### 3.1 Observed wind, geostrophic wind, and thermal wind

**FIGURE 2:**
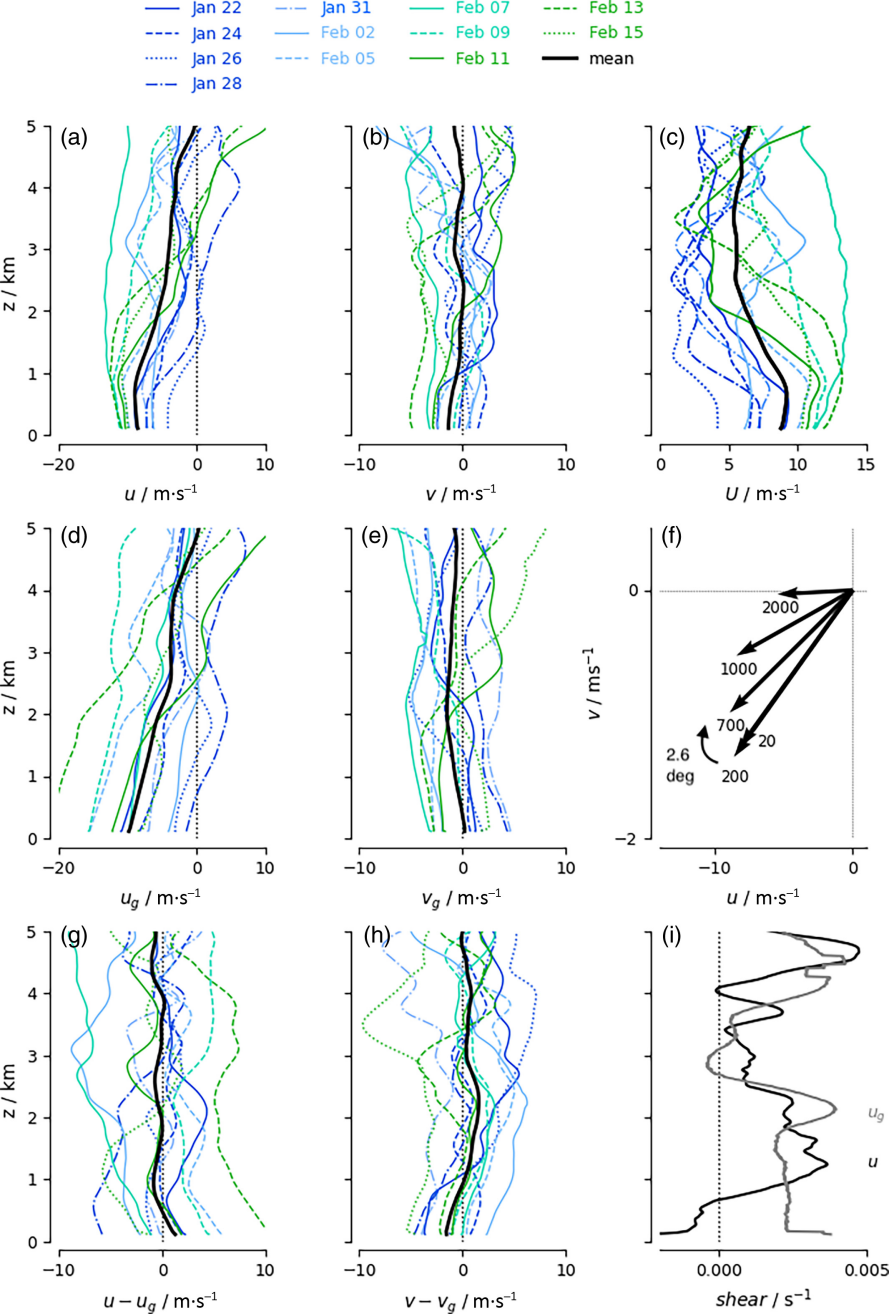
Wind and geostrophic wind profiles for individual HALO flight days (in color) denote the large deviations in wind and mesoscale pressure gradients from the EUREC${}^{4}$A/ATOMIC flight mean (in black). From top to bottom, left to right are shown: the zonal wind u, meridional wind v, wind speed U, geostrophic zonal and meridional wind $u_{g},v_{g}$, wind vectors at selected heights in m (Ekman spiral), geostrophic departures $u-u_{g},v-v_{g}$, and shear in the mean zonal and mean geostrophic zonal wind $\partial_{z}u,\partial_{z}u_{g}$ [Colour figure can be viewed at wileyonlinelibrary.com]

During EUREC${}^{4}$A/ATOMIC, the winds revealed well‐known features of the trade‐wind layer (Malkus, 1958). The profiles of wind speed, zonal wind, and meridional wind, as averages over each HALO flight (color) and averaged over all HALO flights (black), are shown in Figure 2a–c. The mean profile of wind speed was dominated by the zonal wind component, with a (zonal) wind maximum near the mixed‐layer top and cloud base (∼700 m), with winds turning westerly above ∼5 km. The meridional wind was much weaker from the north near the surface and approaching zero above 1 km on average.

**FIGURE 3:**
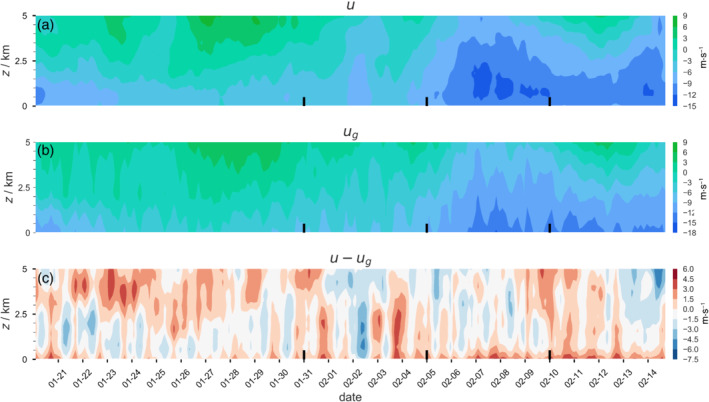
Evolution of u, $u_{g}$, and the geostrophic departure $u-u_{g}$ from ERA5 reanalysis over a 1,000 × 1,000 km${}^{3}$ area, illustrating that, while u follows $u_{g}$ on synoptic time‐scales, significant departures from geostrophy are present on (sub‐)daily time‐scales [Colour figure can be viewed at wileyonlinelibrary.com]

EUREC${}^{4}$A/ATOMIC started out in January with winds that were weaker than average and with strong vertical wind shear above the well‐mixed layer, evident from westerly winds extending down to lower altitudes. These day‐to‐day variations are easier to observe from a time series of u from ERA5 (Figure 3a). February continued with winds that were stronger than average, with weak vertical wind shear in the first week(s) of February. Satellite imagery and flight reports indicate that cloud patterns evolved from frequent popcorn cumuli (“Sugar') in late January to precipitating cumuli organized along cold pools (“Gravel”) and regular appearances of larger cloud clusters in February: either isolated and topped with stratiform veils surrounded by clear skies (“Flowers”) or embedded in large fishbone‐like skeletal cloud structures (“Fish”) (Schulz, 2021). Several days, including HALO flight days (February 2, 5, 7, and 13), exhibited a deep layer of strong easterlies. Towards the end of EUREC${}^{4}$A/ATOMIC, vertical wind shear strengthened again.

The winds are expected to be close to geostrophy at levels where friction vanishes, as baroclinic instability is small in the subtropics. One question we are interested in is whether the zonal winds follow changes in the geostrophic wind closely, or whether advection and friction play a significant role in driving ageostrophic winds.

The zonal and meridional geostrophic winds ($u_{g},v_{g}$) are defined as

(9) $$\begin{array}{ll} u_{g} & =-\frac{1}{f\rho }\partial_{y}p, \end{array}$$

(10) $$\begin{array}{ll} v_{g} & =\frac{1}{f\rho }\partial_{x}p, \end{array}$$

and are derived from the observed circle‐averaged pressure gradients at every height level, using the circle‐averaged air density ρ, and f as the Coriolis parameter at the circle‐averaged latitude. Additionally, $u_{g}$ is derived from ERA5 over a much larger 1,000 × 1,000 km${}^{2}$ area. Averaged over all flights (in black), the departure from geostrophy is less than 1 m·s${}^{-1}$ at all altitudes, with somewhat larger departures near the surface. $u-u_{g}$ is positive near the surface, indicating a weaker than geostrophic easterly wind. As the easterly wind slows towards the surface, it will turn counterclockwise towards the direction of low pressure, establishing v<0 and $v-v_{g}<0$ (as $v_{g}\approx 0$), consistent with Ekman turning. However, because winds are well mixed throughout the lowest kilometer, only 2.6° of wind turning exists between the surface layer and cloud base (Figure 2f, mind the different scales of the u and v axis).

Evidently, winds are much further from geostrophy on individual flights (colored lines in Figure 2), with departures ranging from 0 to over 10 m·s${}^{-1}$ in u and up to 5 m·s${}^{-1}$ in v. On some days, zonal winds are close to geostrophy up to 2 km (e.g., January 24, 26, and 31, and February 11), but may turn negative aloft as the geostrophic wind changes sign above 2 km (e.g., on January 31 and February 11). Several days also exhibit a large negative geostrophic departure (e.g., January 28 and February 2, 7, and 15), which implies the presence of supergeostrophic winds.

The question arises whether the observed changes in geostrophic wind and departures are representative of the ongoing synoptics (advection) or reflect the influence of convection and mesoscale flows. The advection tendency is, on average, an order of magnitude smaller than the pressure gradient, Coriolis, and frictional forces, but it is quite variable from day to day and can accelerate winds (Section 4.2). The geostrophic winds from ERA5 determined over an area approximately 25 times larger are also up to −18 m·s${}^{-1}$ in February, with values for $u-u_{g}$ that vary greatly from day to day (Figure 3c). Some days with very strong negative departures, for example, on February 2, are present in both JOANNE and ERA5, which suggests that they can be synoptically driven, while on other days JOANNE and ERA5 disagree (e.g., February 13). As discussed in Section 4.3, the IFS forecast and ERA5 exhibit wind biases throughout the lower troposphere that are in line with differences in the observed and modeled wind tendencies, suggesting that convection and mesoscale flows may contribute to pressure gradients in a way not captured fully by the model (reanalysis).

The vertical shear in u and $u_{g}$ averaged over all flights is shown in Figure 2i. On the scale of the large‐scale overturning circulation, the vertical shear in the zonal geostrophic wind may be explained by thermal wind, which is defined as

(11) $$\partial_{z}u_{g}∼-\frac{g}{f\;T}\partial_{y}T.$$

The right‐hand side represents the temperature (T) contrast between tropical and subtropical air masses and g is the gravitational constant. The mean shear in $u_{g}$ over HALO flights exhibits a local maximum near 2 km, which is also present in the mean thermal wind determined over a 1,000 × 1,000 km${}^{2}$ area from ERA5 during EUREC${}^{4}$A/ATOMIC (see Figure S2 in the Supplementary Information), except that the latter is smaller in magnitude. Perhaps it is not coincidental that the thermal wind peaks just below the mean trade‐wind inversion (∼2.3 km), as this is where radiative cooling in subtropical boundary layers is pronounced.

The difference between $\partial_{z}u_{g}$ and $\partial_{z}u$ may be interpreted as the efficiency with which smaller‐scale processes modify the large‐scale wind profile. This appears efficient in the mixed layer up to 1 km, where most shear is removed, but inefficient in the lower cloud layer, where u has more shear than $u_{g}$ (and above the trade inversion, but here shear is small anyway). LESs have shown that, by producing countergradient momentum flux in the cloud layer (Dixit *et al*., 2020), convection may help explain the origin and maintenance of the local wind maximum near cloud base.

### 3.2 Wind diurnality

**FIGURE 4:**
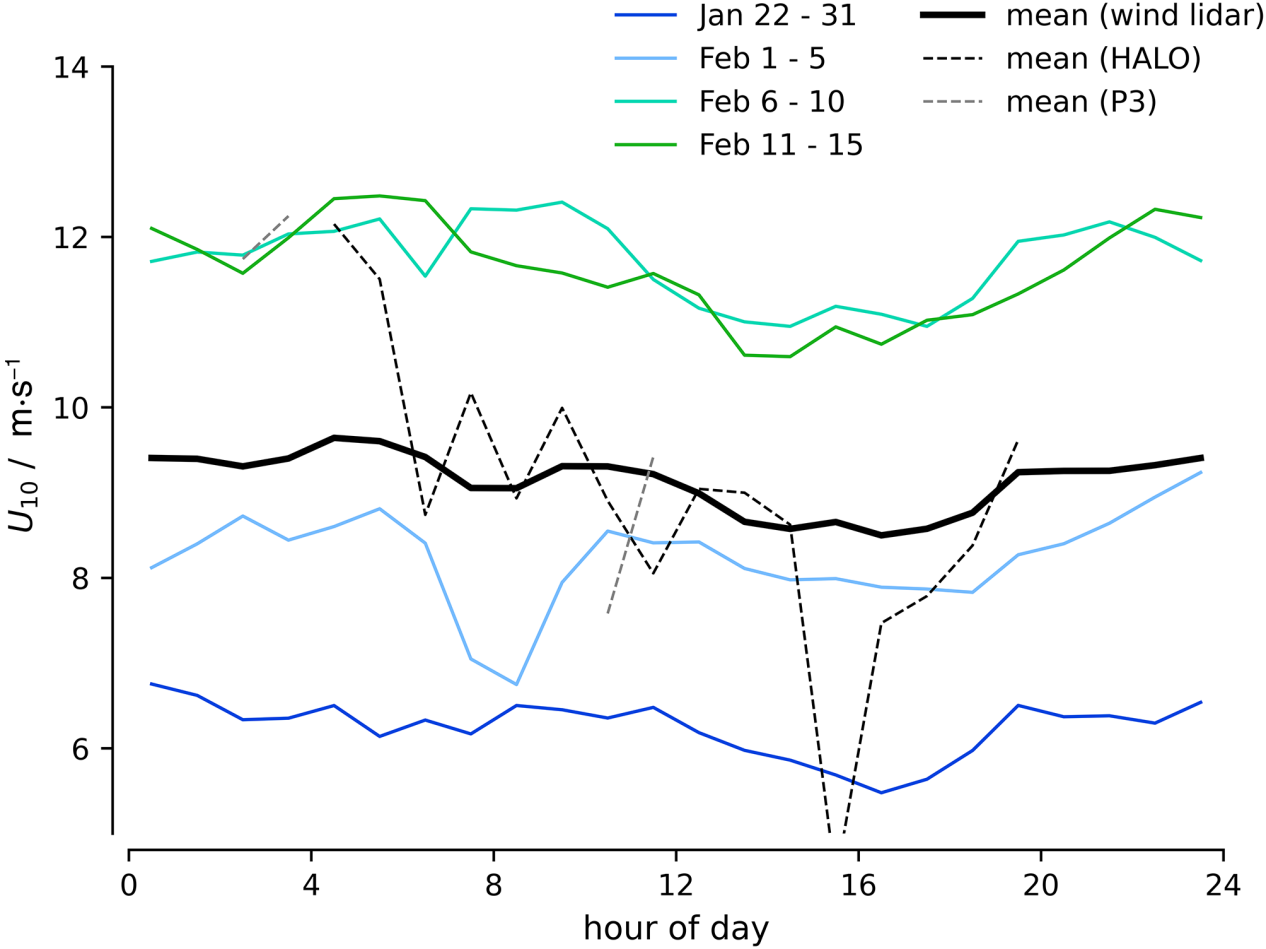
Mean diurnal cycle of 10‐m wind speed over different (synoptic) periods observed by a wind lidar on R/V Meteor. HALO flights generally captured the gradual decline in wind speed from night to day [Colour figure can be viewed at wileyonlinelibrary.com]

The winds during EUREC${}^{4}$A/ATOMIC experienced a diurnal cycle, with weaker winds during the daytime and stronger winds during the night, in line with findings from previous studies (Nuijens *et al*., 2009; Vial *et al*., 2019; 2021). Because many HALO flights started in the (early) morning hours and lasted for about 8 hr, they experienced a gradual decline in wind speed during the flight. Figure 4 shows the composite diurnal cycle of 10‐m wind speed during EUREC${}^{4}$A/ATOMIC in solid black, as observed by a wind lidar situated on board the RV Meteor. The colored lines represent the composite diurnal cycle for four subsequent periods, from January (blue) to mid February (green). Also shown is the composite diurnal cycle of winds observed from the HALO dropsondes (dashed black lines) and P3 dropsondes (grey dashed lines; see the flight timing in Table 1). The mean diurnal cycle from HALO dropsondes is overestimated, because most January flights of HALO sampled the afternoon, while most February flights sampled the morning.

**TABLE 1:** Overview of JOANNE flight circles used in the budget computation: date of flight, aircraft, number of circles, and timing of flight, where *morning* denotes flights between 0500 and 1300 LT, *noon* flights between 0800 and 1600 LT, *afternoon* flights between 1100 and 1900 LT), and *night* flights between 0100 and 0300 LT

Date	Aircraft	Circles	Timing
Jan 17	P3	1	Noon
Jan 19	P3	1	Noon
Jan 23	P3	2	Afternoon
Jan 31	P3	1	Noon
Feb 3	P3	1	Noon
Feb 4	P3	1	Noon
Feb 5	P3	1	Noon
Feb 9	P3	2	Night
Feb 10	P3	2	Night
Feb 11	P3	2	Night
Jan 19	HALO	1	Afternoon
Jan 22	HALO	6	Afternoon
Jan 24	HALO	6	Morning
Jan 26	HALO	6	Noon
Jan 28	HALO	6	Afternoon
Jan 31	HALO	6	Afternoon
Feb 2	HALO	6	Noon
Feb 5	HALO	6	Morning
Feb 7	HALO	6	Noon
Feb 9	HALO	6	Morning
Feb 11	HALO	5	Noon
Feb 13	HALO	6	Morning
Feb 15	HALO	5	Afternoon

Savazzi *et al*. (2021) studied the diurnality of wind and the momentum budget in the IFS model and in ERA5 and show that the pressure gradient force reaches a minimum between 1100 and 1700 LT, consistent with the presence of weak winds during the daytime. It then increases in strength rapidly to reach its largest values between 1700 and 2100 LT, afterwards reducing slowly during the night and morning. Previous studies have shown that diurnal and semidiurnal variations in pressure gradients over (sub)tropical oceans are pronounced (Deser, 1993; Rei and Clara, 2008). They can be related to atmospheric thermal tides, diurnal variations in SST and deep convection (over land and ocean), and related subsidence waves (Wood *et al*., 2009). The diurnality observed east of Barbados is not well understood, but we hypothesize that diurnality in the strength of the Hadley cell is an important driving force. An increase in pressure gradient force during the afternoon may relate to the development of lower surface pressures over the nearby continent (South America), as deep convection over land peaks in the afternoon. During the night and early morning, a peak in deep convection over the tropical oceans may also help maintain large pressure gradients.

The diurnality in local convection may also play a role: while the pressure gradient force begins to decrease slowly after 2100 LT, wind speeds only reach their maximum in the early morning. The presence of deeper precipitating clouds during the night that are maximized just before sunrise (Vial *et al*., 2019) could help delay the decrease in near‐surface winds. Whichever is the driving mechanism, if we ignore the local change in wind during flights, we wouldobtain a weaker eddy momentum flux divergence. In the next section, we derive and explain the observed momentum budget in detail.

## 4 THE HORIZONTAL MOMENTUM BUDGET

We analyze the momentum budget in two ways, each having their advantage. First, we explore the budget of the zonal and meridional wind, which we can compare with the ECMWF IFS wind budget, which is derived for the exact same area and flight times. We then transform the winds into a natural coordinate system aligned with the direction of the mean wind, which shows the forces that drive wind speed and wind turning more naturally.

### 4.1 Zonal and meridional wind budget

The momentum budget of the circle‐mean horizontal wind, denoted by an overbar, can be written as

(12) $$\begin{array}{ll} \partial_{t}ū+\bar{u}\cdot \nabla u & =-{\bar{\rho }}^{-1}\partial_{x}p+f\bar{v}+{ℱ}_{u}, \end{array}$$

(13) $$\begin{array}{ll} \partial_{t}\bar{v}+\bar{u}\cdot \nabla v & =-{\bar{\rho }}^{-1}\partial_{y}p-fū+{ℱ}_{v}, \end{array}$$

where the left‐hand side represents the local storage (tendency) term and horizontal and vertical advection by the circle‐mean wind. The first term on the right‐hand side represents the pressure gradient force and the second term the Coriolis force, with f as the Coriolis parameter. ${ℱ}_{u},{ℱ}_{v}$ represent all processes within the circle that would accelerate the zonal and meridional flow. They may be interpreted as an eddy momentum flux convergence:

(14) $$\begin{array}{ll} {ℱ}_{u} & \equiv -\frac{\partial \bar{u^{\prime }u^{\prime }}}{\partial x}-\frac{\partial \bar{u^{\prime }v^{\prime }}}{\partial y}-\frac{\partial \bar{u^{\prime }w^{\prime }}}{\partial z}, \end{array}$$

(15) $$\begin{array}{ll} {ℱ}_{v} & \equiv -\frac{\partial \bar{v^{\prime }u^{\prime }}}{\partial x}-\frac{\partial \bar{v^{\prime }v^{\prime }}}{\partial y}-\frac{\partial \bar{v^{\prime }w^{\prime }}}{\partial z}, \end{array}$$

where the overbar indicates the mean over an area encompassed by the circle and primes indicate perturbations from the mean flow over the circle. It is typically assumed that vertical eddy transport dominates over horizontal eddy transport (an assumption we come back to in Section 6), and making use of Equations 9 and 10, which combine the pressure gradient and Coriolis term into a geostrophic departure term, the budgets may be written as

(16) $$\begin{array}{ll} \partial_{t}ū+\bar{u}\cdot \nabla u & \approx f(\bar{v}-{\bar{v}}_{g})-\frac{\partial \bar{u^{\prime }w^{\prime }}}{\partial z}, \end{array}$$

(17) $$\begin{array}{ll} \partial_{t}\bar{v}+\bar{u}\cdot \nabla v & \approx -f(ū-{ū}_{g})-\frac{\partial \bar{v^{\prime }w^{\prime }}}{\partial z}. \end{array}$$

Each circular dropsonde array provides the geostrophic departure term. The horizontal advection terms are calculated by multiplying the zonal and meridional wind gradient (Equation 1) with the circle‐mean horizontal winds ($ū,\bar{v}$), and the vertical advection terms by multiplying the circle‐mean vertical wind $\bar{w}$ (Equation 3) with the vertical gradient of $ū,\bar{v}$. The change in wind is fairly linear over the course of each flight and $\partial_{t}ū$ and $\partial_{t}\bar{v}$ can be determined as the difference in $ū,\bar{v}$ between the last‐ and first‐flown circle, or as a linear regression of circle‐mean winds. This tendency is combined with the forcing terms averaged over all 7–8 circles to give flight‐mean residuals ${ℱ}_{u}$ and ${ℱ}_{v}$.

### 4.2 Sampling uncertainty

**FIGURE 5:**
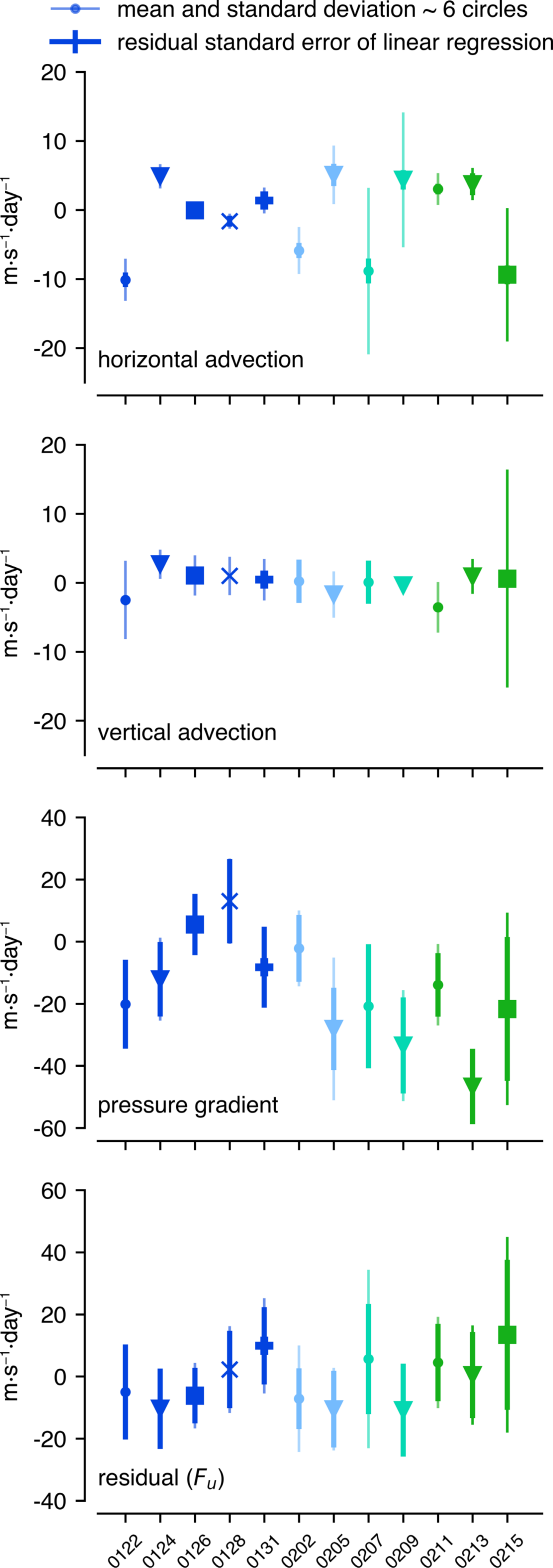
Large‐scale forcings and residual for the zonal wind component at 200 m derived from JOANNE. Markers denote the flight‐mean, thin lines denote the standard deviation across all circles of each flight, and the thick vertical line denotes the mean of the residual standard error of the linear regression (Equation 2), taken as the square root of individual errors squared. For the residual, the standard errors of the advection and pressure gradient are combined [Colour figure can be viewed at wileyonlinelibrary.com]

The horizontal and vertical advection of zonal wind, the corresponding pressure gradient, and the derived residual (${ℱ}_{u}$) at 200 m are shown as averages for each flight day in Figure 5. The thick vertical bar corresponds to ± the mean residual standard error, which measures the validity of assuming stationarity and linearity in the measured field (Section 2.1). The thin vertical bar represents the standard deviation measured during the ∼6 circles of each flight, which typically spanned 7–8 hr. Compared with the pressure gradient, variability is relatively small for horizontal and vertical advection across the days, with some exceptions (e.g., February 7, 9, and 15). The pressure gradient is more irregular of nature within the circle and also undergoes a considerable diurnal cycle that invalidates the assumption of stationarity (Savazzi *et al*., 2021). In Figure S1 in the Supplementary Information, the zonal and meridional pressure gradients from JOANNE are compared with ERA5 at the circle scale and at a larger scale of 1,000 × 1,000 km${}^{2}$ for all flight days and for February 5 and 13. On average, the observed pressure gradients match those from ERA5 in the circle and on larger scales, especially in the mixed layer and in the meridional direction that drives $u_{g}$. However, on February 5 and 13 the circle‐derived pressure gradients are considerably larger for JOANNE.

While individual circles measure across nature's rich variability, the change in mean flight forcing from one day to the next often exceeds one standard error. Hence, we interpret the day‐to‐day variations as a realistic representation of changes in the prevailing flow field, clouds, and their mesoscale organization. As we discuss next, averaging days with varying mesoscale flows leads to a mean budget that reflects our theoretical understanding and is in line with the operational forecast of the IFS.

### 4.3 Observed versus modeled momentum budget

**FIGURE 6:**
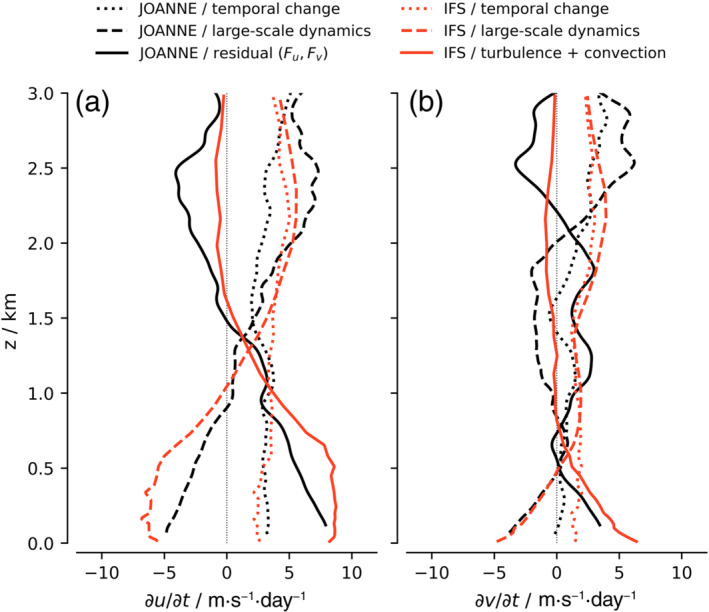
Comparison of the observed momentum budget averaged over all flights with the IFS momentum budget at matching locations and times shows agreement on the depth of the frictional layer in u and v inferred from the residual (JOANNE, solid black line) and from the IFS turbulence and convection tendencies (solid red line). However, differences in the forcing are also present, especially above 1.5 km and near cloud base (in u) [Colour figure can be viewed at wileyonlinelibrary.com]

The budget terms, averaged over all EUREC${}^{4}$A/ATOMIC flights, are compared with the IFS in Figure 6, where the IFS output is extracted at the exact same locations and times as the dropsonde arrays. For both JOANNE and the IFS, the horizontal and vertical advection, pressure gradient, and Coriolis terms are combined and plotted as one “large‐scale dynamical” forcing. The advection term is dominated by the horizontal advection (Figure 5) and is on average smaller than the combined pressure gradient and Coriolis force. The residual in JOANNE (${ℱ}_{u}$ and ${ℱ}_{v}$) is interpreted as the friction produced by turbulence and convection and compared with the parameterized momentum tendencies in the IFS from the turbulence and shallow convection schemes.

The observations and the IFS show remarkable agreement in the overall magnitude of the forcing terms, providing confidence on the one hand in the ability of the observations to derive dynamical tendencies, and on the other hand in the ability of the model to predict the dynamics at the mesoscale. The imbalance between large‐scale dynamical forcing and friction implies a temporal weakening of the zonal and meridional wind during flight hours. The observations and the IFS agree on the temporal change in ū at levels below 1.5 km and on the height where the dynamical forcing of ū changes sign.

The frictional layer, where ${ℱ}_{u}>0$ (a deceleration of the easterly wind), is approximately 1.5 km deep in both the observations and the IFS. The residual in the meridional wind budget should not be overlooked: ${ℱ}_{v}>0$ in the mixed layer up to 500 m (as well as in the cloud layer between 1 and 2.2 km). This corresponds to a weakening of the mean northerly flow. ${ℱ}_{v}$ is more than half the magnitude of ${ℱ}_{u}$ near the surface. Considering the small magnitude of the v− wind compared with the u− wind (Figure 2a,b), ${ℱ}_{v}$ is proportionally large and opposes Ekman wind turning near the surface. Efficient vertical transport might explain the relatively weak wind turning in the lower atmosphere (Figure 2f).

The most prominent differences between the observations and the IFS are observed in (*i*) the u tendencies in the lowest 1 km, with differences maximized near cloud base (∼700 m), (*ii*) ${ℱ}_{u}$ above 1.5 km, (*iii*) the frictional forces in v below cloud base, and (*iv*) the large‐scale dynamical tendency of v in the cloud layer between 700 m and 3 km. Savazzi *et al*. (2021) investigates these differences in detail and shows that they are consistent with biases in the wind profile. For instance, u tendencies from parameterized momentum transport in the IFS are much closer to zero above 1.5 km, which is consistent with too weak easterly winds in the IFS. The negative ${ℱ}_{u}$ above 1.5 km in JOANNE suggests that processes accelerate the easterly wind (winds are easterly winds up to at least 3 km on most days, see Figure 2a). The mean trade inversion during HALO flights was ∼2.26 km, which is about the height where the residual starts to waver back to zero. We hypothesize that convection plays a role in driving stronger easterly flow in the upper cloud layer. Although we cannot rule out other possible sources, for example, gravity waves or errors in the retrieved observed tendencies, ongoing analysis of supporting LESs shows a similar acceleration near cloud tops at times of vigorous convection.

The differences in v tendencies (*iii,iv*) imply that the IFS has a positive tendency in $\bar{v}$ throughout the lowest two kilometers, which is also in line with a too weak meridional wind in the IFS throughout the lower atmosphere (Savazzi *et al*. 2021).

### 4.4 The horizontal wind budget in natural coordinates

To bring out the forcing balance better, the winds are rotated into a natural coordinate system (s,n), in which the s‐axis points in the direction of the wind vector at each height, while the n‐axis is defined positive to the left of the s‐axis. The momentum budget of the circle‐mean wind may then be written as

(18) $$\begin{array}{ll} D{ū}_{s}/Dt & =-{\bar{\rho }}^{-1}\partial_{s}p+{ℱ}_{s}, \end{array}$$

(19) $$\begin{array}{ll} 0 & \approx -{\bar{\rho }}^{-1}\partial_{n}p-f{ū}_{s}+{ℱ}_{n}. \end{array}$$

Because ${ū}_{n}=0$ at each height, ${ū}_{s}$ essentially equals the total wind speed (U, in Figure 2c). The Coriolis force vanishes in the s‐direction, and the advection includes both speed and directional convergence from flow at some angle to the s‐axis. In the n‐direction, $\partial_{t}{ū}_{n}$ vanishes and we assume the centripetal acceleration due to curving of the flow ($U^{2}/R$) to be small. If ${ℱ}_{n}\neq 0$, eddy momentum flux divergence is turning the wind. Assuming that s is directed purely west in the case of an easterly trade wind and n is directed to the south, ${ℱ}_{n}>0$ implies that wind is “backing” (e.g., turned counterclockwise, towards the low‐pressure ITCZ in the south), consistent with the Ekman spiral. ${ℱ}_{n}<0$ implies that wind is “veering” (e.g., turned clockwise, towards the north away from the low‐pressure region).

**FIGURE 7:**
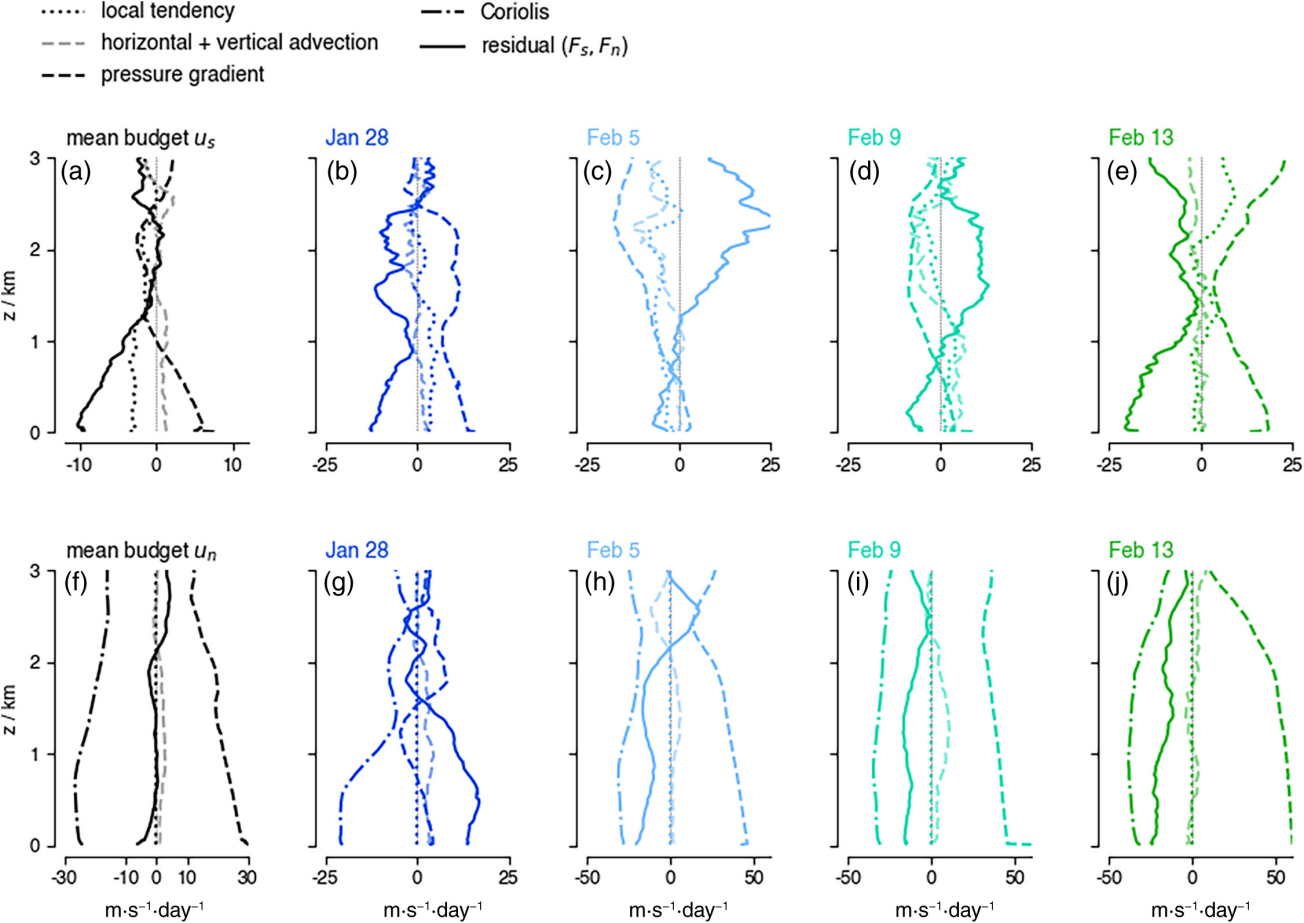
The pressure gradient and inferred frictional force can differ substantially (and even reverse sign) from day to day, as illustrated by the along‐wind ($u_{s}$) and cross‐wind ($u_{n}$) momentum tendencies averaged over all flights (black), or flights on January 28 (blue), February 5 (light blue), February 9 (aquamarine), and February 13 (green). The averages are calculated over individual profiles that are first aligned with the wind at every height level [Colour figure can be viewed at wileyonlinelibrary.com]

The budgets of $u_{s}$ and $u_{n}$ averaged over all flights are shown in Figure 7 (in black). The budget of ${ū}_{s}$ is similar to that of ū below 1.5 km (approximately the depth of the frictional layer, e.g., Figure 6). Because $u_{s}$ is always positive in the natural coordinate system, ${ℱ}_{s}<0$ (in solid black) implies a frictional force. To first order, ${ℱ}_{s}$ is balanced by the along‐wind pressure gradient. However, unlike in the ū budget, the budget terms are approximately zero above 1.5 km. In other words, there is little pressure or frictional force in the prevailing wind direction at these levels. The nonzero forcing terms in the $\bar{u}$ budget thus reflect that the zonal component of the wind generally weakens with height above ∼1 km (see Figure 2a), which causes a backing of the wind with height (when v<0) or a veering of the wind (when v>0). ${ℱ}_{u}>0$ above 1.5 km implies that processes are acting to turn the wind by increasing the easterly wind component.

**FIGURE 8:**
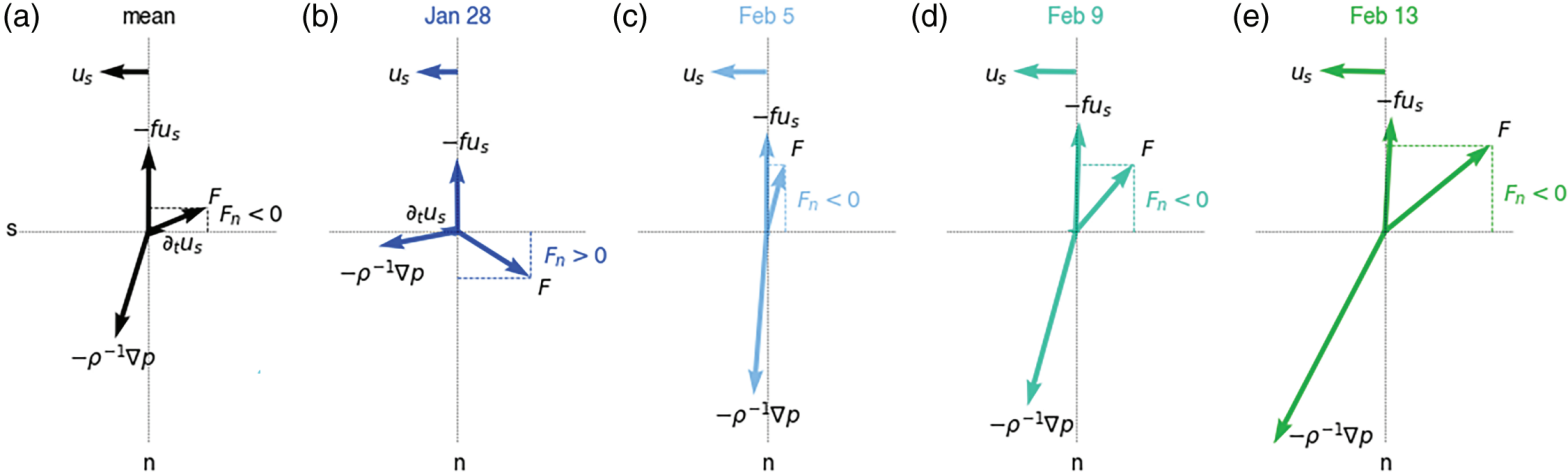
Wind‐vector balance at 20 m in natural coordinates (s,n) illustrates that, on average, the inferred frictional force (ℱ) vector is not pointing in the opposite direction of the prevailing flow ($u_{s}$), but at an angle created by a cross‐wind frictional component directed to the right of the flow (${ℱ}_{n}<0$), which points in the same direction as the Coriolis force. On February 5, the presence of large ${ℱ}_{n}<0$ and little along‐wind friction (${ℱ}_{s}\approx 0$) leads to flow that is almost parallel to the isobars[Colour figure can be viewed at wileyonlinelibrary.com]

The mean budget of $u_{n}$ includes a large pressure gradient in the cross‐wind direction, which is largely balanced by the Coriolis force (the dash–dotted black line, Figure 7f). A veering of the wind is caused by the residual (${ℱ}_{n}<0$) in the mixed layer (<500 m) and in a layer between 1.5 and 2 km (note that ${ℱ}_{n}$ and ${ℱ}_{s}$ have comparable magnitudes near the surface, but the axes are scaled differently). In the cross‐wind direction, subcircle scale processes act to oppose cross‐isobaric wind turning. The vector balance near the surface, shown in Figure 8a, illustrates that the total pressure gradient force is almost aligned with n, which implies that the flow is almost parallel to the isobars. The pressure gradient force is balanced by the combined Coriolis force and ℱ, which has a significant component to the right of the flow ${ℱ}_{n}<0$. In the next section, we will show how this mean balance is established on individual days.

### 4.5 Day‐to‐day variability

**FIGURE 9:**
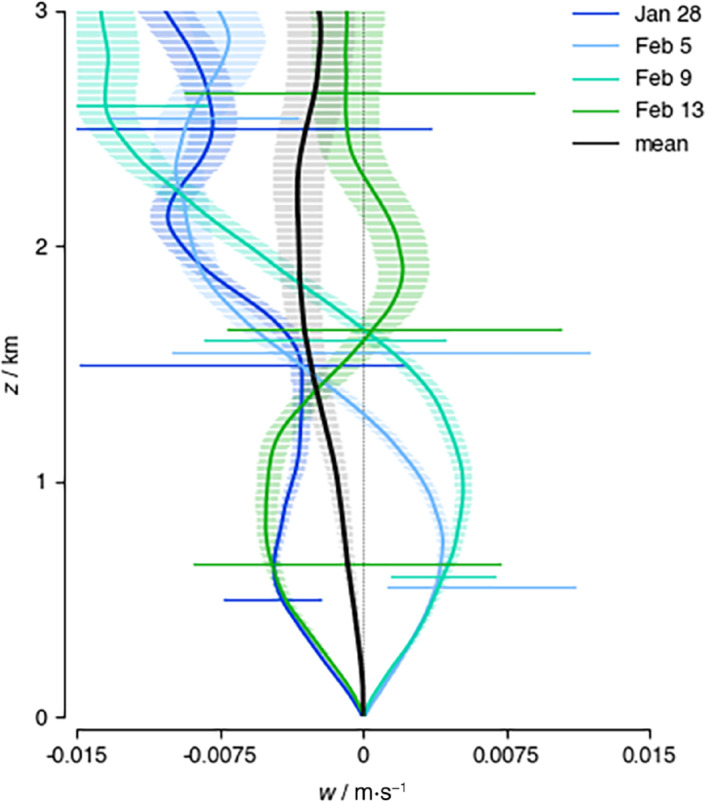
Flight‐mean vertical velocity profile on January 28, February 5, February 9, and February 13, and as the EUREC${}^{4}$A/ATOMIC flight mean (in black). The shaded area corresponds to the mean residual standard error (Equation 2) and horizontal lines at selected heights span the minimum and maximum w encountered on a flight. On February 5 and February 9, mean rising motion at low levels and descending motion aloft coincide with the layer of positive ${ℱ}_{s}$ above z = 1.5 km seen in Figure 7c,d [Colour figure can be viewed at wileyonlinelibrary.com]

As the winds strengthened from January to February (Figure 3), convection became more vigorous and larger cloud structures developed. The flights encountered mostly shallow “Sugar” clouds on January 28, while on February 5 and 9 they encountered larger cloud aggregations with stratiform outflow and isolated cumulus towers pushing through the inversion and with strongly sheared cloud tops and strong rain echoes. On February 13, the circles captured part of a ‘Fish’ (Figure 1). As highlighted in George *et al*. (2021a), the mesoscale variability in divergence is large and reflected in the range of vertical motion encountered on individual days (Figure 9). January 28 and February 13 experienced mean divergence below cloud base (Figure 9), which on February 13 turned to convergence above 1.5 km, reflecting a shallow circulation associated with the nearby fish. In contrast, most circles on February 5 and 9 measured the ascending branches of mesoscale circulations with convergence below cloud base and divergence in the cloud layer.

The budget profiles and near‐surface wind vector balance for these four representative days are shown in Figures 7b–e and 8b–e (a complete time series of the budget components is included in Figure S4 in the Supporting Information). The stronger winds and deeper cloud field were associated with an increase in pressure gradient force in the direction perpendicular to the flow (Figure 7h–j, dashed lines). The diurnal cycle in the meridional pressure gradient may play a role here, because days with a large positive dynamical forcing, consistent with southward winds strengthening throughout the (early) morning, are morning flights (February 5, 9, 13). However, the early February days (5–9) experienced a much smaller along‐wind pressure gradient that changed sign in the cloud layer. Figure 7c,d also reveals that these days had much smaller ${ℱ}_{s}$ near the surface, and comparably larger and negative ${ℱ}_{n}$ (Figure S4). Furthermore, a layer of positive ${ℱ}_{s}$ is present above z = 1.5 km, coinciding with the layer of divergence (Figure 9).

The vector balance in Figure 8c,d shows that, while the flow on January 28 is directed towards the region of lowest pressure and ${ℱ}_{n}>0$, on February 5 and 9 the pressure gradient is balanced by the Coriolis force and by ℱ, such that the flow is almost parallel to the isobars. ℱ has a relatively large negative ${ℱ}_{n}$ component, which would tend to reduce cross‐isobaric flow and Ekman pumping within the ITCZ.

Assuming that ℱ represents turbulence and convection within the circles, the question arises whether convection could facilitate the observed reduction in ${ℱ}_{s}$. Convective plumes can accelerate the flow by removing air that has slowed down near the surface (and gained a westerly component if the flow were pure easterly). Air with larger momentum may also be introduced through dry or precipitation downdrafts (Helfer *et al*., 2020; Saggiorato *et al*., 2020). An eddy momentum flux convergence carried by convective circulations that accelerate the easterly wind may in such a case compensate for the eddy momentum flux divergence carried by smaller turbulence that slows down the easterly wind. In the cloud layer and near the inversion, an eddy momentum flux convergence associated with detrainment and precipitating downdrafts may also have contributed to an acceleration of the flow (${ℱ}_{s}>0$). It is these heights where the IFS and JOANNE u budgets differ most, and, as we mentioned in Section 4.3, ongoing work using LESs shows a similar acceleration near cloud tops driven by convection.

## 5 EDDY MOMENTUM FLUX PROFILES

To obtain a profile of the eddy momentum flux, we can integrate the residual,

(20) $$\begin{array}{ll} {ℱ}_{s^{\prime }} & \approx -\frac{\partial \bar{u_{s^{\prime }}^{\prime }w^{\prime }}}{\partial z}\equiv \frac{1}{\rho }\frac{\partial \tau_{s^{\prime }}}{\partial z}, \end{array}$$

(21) $$\begin{array}{ll} {ℱ}_{n^{\prime }} & \approx -\frac{\partial \bar{u_{n^{\prime }}^{\prime }w^{\prime }}}{\partial z}\equiv \frac{1}{\rho }\frac{\partial \tau_{n^{\prime }}}{\partial z}, \end{array}$$

along the vertical height axis. The apostrophe notation ($s^{\prime },n^{\prime }$) indicates that winds and tendencies are first transformed into a shared coordinate frame that is aligned with the wind closest to the surface, which is 10 m in the dropsonde observations (note that this is different from the alignment with wind at each respective height level used earlier to bring out the forcing balance at each height).

**FIGURE 10:**
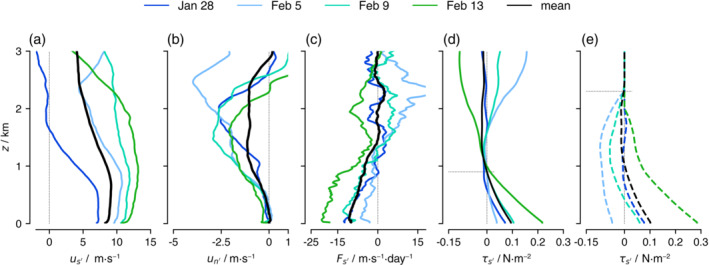
Profiles of (a) $u_{s^{\prime }}$, (b) $u_{n^{\prime }}$, (c) ${ℱ}_{s^{\prime }}$, and eddy momentum flux $\tau_{s^{\prime }}$ are shown for selected days, whereby the eddy momentum flux is derived using either the assumption of zero flux at the local wind maximum (∼900 m on average, denoted by the thin dotted horizontal line in (d)), or assuming a vanishing flux above the trade inversion (∼2.3 km on average, thin dotted line in (e)). All winds are first transformed into a natural coordinate frame that is aligned with the 10‐m wind. The mean $\tau_{s^{\prime }}$ profiles (in black) in (d) and (e) are very similar, which suggests both assumptions are valid [Colour figure can be viewed at wileyonlinelibrary.com]

**FIGURE 11:**
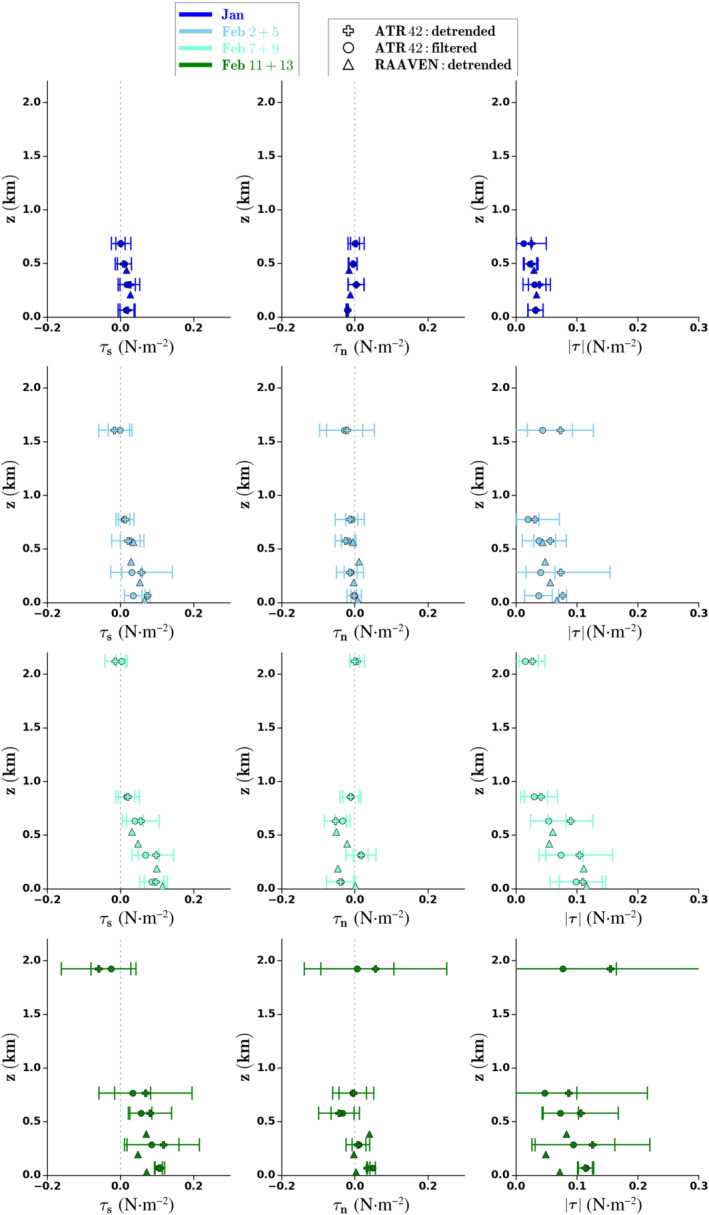
Along‐wind ($\tau_{s}$), cross‐wind ($\tau_{n}$), and total momentum flux (τ) profiles measured on board the ATR and RAAVEN show large momentum fluxes in the mixed layer (∼250 m) and near cloud tops (>1.5 km) with large spatial variability (especially on February 11 and 13) denoted by the horizontal bars, which represent the range of ATR leg means at a given height. While the filtered moments (circles) are representative of typical turbulence, the detrended moments (crosses and triangles) include the contribution of mesoscale fluctuations (between 5 and 30 km for the ATR and up to 5 km for the RAAVEN) [Colour figure can be viewed at wileyonlinelibrary.com]

Performing the vertical integration requires a boundary assumption on $\tau_{s^{\prime }},\tau_{n^{\prime }}$. In Holland and Rasmusson (1973) and Brümmer *et al*. (1974) it was assumed first that the surface momentum flux (surface stress) is directed opposite to the surface wind, so that $\tau_{n^{\prime }}$ = 0 at the surface. As we shall see in Figure 11b,e,h, this is not a bad assumption. Second, it was assumed that the along‐wind momentum flux at the height of the local wind maximum is zero ($\tau_{s^{\prime }}=0$ where $\partial_{z}u_{s^{\prime }}=0$). This assumption is used to construct the profile of $\tau_{s^{\prime }}$ for individual days and the overall mean (solid lines in Figure 10d). In addition, the profile is derived assuming that the flux vanishes at the trade inversion of each selected day (dashed lines in Figure 10e). For reference, Figure 10a–c shows the profiles of $u_{s^{\prime }}$ and $u_{n^{\prime }}$ as averages over all flights (in black) and for the individual days exemplified before (in blue/green), and the same for ${ℱ}_{s^{\prime }}$. By aligning all winds with the 10‐m wind, the profile of $u_{n^{\prime }}$ can become nonzero. Its negative value gained with height implies a wind veering with respect to the 10‐m wind. The profile of ${ℱ}_{s^{\prime }}$ is similar, but not the same as the profile of ${ℱ}_{s}$ in Figure 7a, because of the different coordinate frame transformation. While ${ℱ}_{s}$ represents, at each height, the friction in the prevailing wind direction, ${ℱ}_{s^{\prime }}$ represents only the frictional force experienced in the direction of the near‐surface wind.

While the two assumptions lead to very different flux profiles on individual days, the profiles of mean $\tau_{s^{\prime }}$ in solid and dashed black are almost identical, which suggests both assumptions are valid on average. At 10 m, $\tau_{s^{\prime }}$ is ∼0.1 N·m${}^{-2}$, which is not an unreasonable value. The small negative values observed in the cloud layer above the wind maximum are consistent with countergradient momentum transport in simulations (Larson *et al*., 2019; Dixit *et al*., 2020; Helfer *et al*., 2020), whereby upward transport carries faster momentum from the local wind maximum ($u_{s}^{\prime }>0$, $w^{\prime }>0$, $\bar{u_{s}^{\prime }w^{\prime }}>0$) against the wind gradient in the cloud layer ($\partial_{z}u_{s}<0$). The cross‐wind momentum flux is not shown. Because ${ℱ}_{n^{\prime }}$ is nonzero, $\tau_{n^{\prime }}$ would attain considerable values throughout the mixed layer. For instance, $\tau_{n^{\prime }}\approx$ 0.04 N·m${}^{-2}$ at 1 km and 0.08 N·m${}^{-2}$ at 2.3 km. These values are 40% (respectively 80%) of $\tau_{s^{\prime }}$ at the surface, which suggests the presence of considerable cross‐wind eddy momentum flux.

The ATR and RAAVEN momentum flux profiles offer the opportunity to evaluate the assumptions of where momentum fluxes vanish. They also reveal information on the magnitude of the cross‐wind fluxes and the influence of mesoscale wind fluctuations. Furthermore, their daily variations can be compared with flux variations derived from the budget (Section 4.2).

The along‐wind, cross‐wind and total momentum fluxes measured on board the ATR and the RAAVEN are shown in Figure 11 for four groups of days in January and February. The turbulent fluxes from the ATR are calculated per leg from either a detrended series (whose mean is denoted by the plus marker) or a high‐pass‐filtered series with a cutoff wavelength of ∼5 km (denoted with a circle marker). The high‐pass filter removes the contribution of mesoscale features, which will generate a systematic error that reflects the loss of information, but the filtering will reduce the random error generated by the finite length of the sample. The gain in accuracy in terms of random error compensates significantly for the introduction of a systematic error. While the filtered moments are representative of typical turbulence, the detrended moments include the contribution of mesoscale fluctuations (between 5 and 30 km). The horizontal bars represent the range of leg means at a given height and are a measure of the spatial variability encountered during the flight. The turbulent fluxes for the RAAVEN (triangles) are derived for legs of 3–5 km flown back and forth and are detrended.

There is good agreement between the two datasets, whereby the RAAVEN estimates are typically within the range of values encountered by the ATR. Because the RAAVEN typically flew only a few kilometers away from the coast, while the ATR flew within the HALO circle, the agreement may be less on days with substantial spatial variability, as seen from the ATR legs on, for example, February 11 and 13.

Spatial variability and mesoscale fluctuations on scales between 5 and 30 km are not unimportant. A few examples of where the detrended estimates are larger than the filtered estimates are the lowest legs on February 2 and 5, where the detrended ATR $\tau_{s}$ is almost twice that of the filtered ATR $\tau_{s}$ (Figure 11a,c), the legs at ∼250 m and 700 m on February 7 and 9 (Figure 11d,f), and the highest legs on February 2 and 5 and February 11 and 13. The range in flux is typically largest in the mixed layer and near cloud tops. In particular, the cross‐wind flux ($\tau_{n}$) and the total flux (τ) can be just as large near cloud tops as in the mixed layer: for example, on February 2 and 5 (blue) and on February 11 and 13 (green). Both the ATR and RAAVEN data show that $\tau_{s}$ approaches zero towards 1 km, but is not exactly zero. Hence, the assumption of zero flux near the wind maximum or near cloud tops is not valid, particularly not on February 2 and 5 and February 11 and 13, when $\tau_{s^{\prime }}$ derived from JOANNE approaches values larger than 0.2 N·m${}^{-2}$, while the ATR and RAAVEN suggest values closer to 0.1 N·m${}^{-2}$.

**FIGURE 12:**
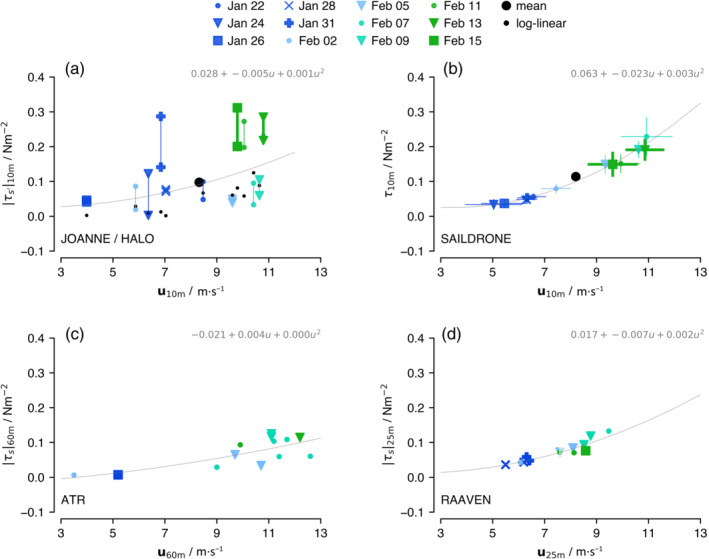
The 10‐m momentum flux is plotted against 10‐m wind speed for each HALO flight (day) using different observations: (a) Fluxes retrieved from the JOANNE momentum budget, where the two estimates connected by a vertical line correspond to the two different assumptions used in deriving the flux (see Figure 10d,e); (b) the 5‐min momentum flux from the Saildrone measurements derived using the COARE3.6 bulk algorithm, with bars denoting one standard deviation; (c) the eddy‐covariance flux measurements from the ATR at the lowest flight legs ∼60 m; and (d) the eddy‐covariance flux measurements from the RAAVEN at the lowest flight legs ∼25 m. The small black dots in (a) correspond to τ derived by fitting an assumed log–linear profile to the flight‐mean wind profiles from JOANNE. The thin grey line represents a second‐order fit[Colour figure can be viewed at wileyonlinelibrary.com]

Both the ATR and the RAAVEN indicate a general increase in momentum flux from January to mid‐February, as expected with a strengthening of the winds, assuming that momentum fluxes are produced predominantly by shear‐driven turbulence, which is in line with the budget‐derived flux profiles (Figure 10d,e). The near‐surface momentum fluxes are also plotted against wind speed for individual flights days in Figure 12. Figure 12a shows the fluxes derived from the JOANNE momentum budget (Figure 10d,e), whereby the vertical line spans the fluxes derived using zero flux either near the wind maximum of near the trade inversion. To provide some reference of how turbulent momentum fluxes may scale with wind speed, the small black circles in Figure 12a show τ derived by fitting an assumed log–linear profile consistent with Monin–Obukhov log‐layer theory to flight‐mean along‐wind profiles in the surface layer (between z = 10 and 200 m):

(22) $$\tau =\tau_{s}=\rho u{*}^{2}=\rho \kappa^{2}\;{\left(\frac{du_{s}}{d\ln z}\right)}^{2},$$

using κ=0.4. On most days, τ derived assuming a log‐linear wind profile is on the lower end of the JOANNE estimate, with an average u∗=0.2 m·s${}^{-1}$ at $u_{s}=8.3$ m·s${}^{-1}$ compared with u∗=0.29 m·s${}^{-1}$ from JOANNE, which is not surprising for an unstable convective boundary layer. Also shown in Figure 12b are the 5‐min momentum fluxes from the Saildrone measurements derived using the COARE3.6 bulk algorithm, whereby the vertical and horizontal bars denote one standard deviation. The Saildrone typically cruised an area somewhat further west, except for February 13 and 15 when it was near the circle (denoted with somewhat thicker markers/lines). The Saildrone suggests a more rapid pickup of τ with wind speed. In comparison, the JOANNE fluxes on January 31 and February 11 and 13 jump out as being relatively large, while JOANNE fluxes on February 5, 7, and 9 are relatively small.

The eddy‐covariance fluxes from the ATR's lowest flight legs ∼60 m and from the RAAVEN's lowest flight legs ∼25 m are shown in Figure 12c,d. These measurements are at a higher altitude, which likely explains the slower pickup of $\tau_{s}$ with wind speed. The RAAVEN illustrates that spatial variability is not unimportant, as its data collected at a location closer to Barbados are shifted to lower wind speeds. Despite the limitations of a comparison, the ATR fluxes suggest that τ was indeed relatively low during February 5, 7, and 9, as suggested by JOANNE's fluxes, while the JOANNE fluxes on January 31 and February 11–15 are likely overestimated.

## 6 DISCUSSION

Our motivation for comparing eddy momentum fluxes is to evaluate our assumptions and interpretation of the budget residual as a friction established by vertical eddy momentum flux divergence. We find that the JOANNE τ estimates differ much more from flight to flight than the in situ measurements. One plausible explanation is that we wrongly assume the height at which the flux goes to zero, which is especially difficult to pinpoint on days when the trade inversion or local wind maximum are poorly defined, such as on February 13 (Figure 10a in green), which has large momentum fluxes between 0.5 and 1 km and near 2 km (Figure 11g).

Another reason may be that the observed momentum fluxes include different scales of variability. The budget leads to an estimate of the friction produced by momentum fluxes within the circle (∼220 km), which includes meso‐alpha (2–20 km) and meso‐beta scales (20–200 km). The in situ momentum fluxes include fluctuations generally on scales <5 km (Saildrone, RAAVEN) and <20–30 km (ATR). Unlike thermodynamic perturbations, which are inherently well correlated with up‐ and downdrafts, horizontal momentum and vertical velocity are less correlated, especially when pressure gradients also play a role. Even the sign of the momentum flux is sensitive to the inclusion of different scales of eddies (Zhu, 2015).

The presence of mesoscale variability, as also suggested by large spatial variations in the fluxes measured by the ATR (Figure 11), brings into question the assumption of negligible horizontal eddy transport. The assumption of horizontally homogeneous flow used in Equation 17 (e.g., momentum fluxes leaving and entering the circle are the same) is then not valid:

(23) $$\begin{array}{ll} -\;\frac{\partial \bar{u^{\prime }u^{\prime }}}{\partial x}-\frac{\partial \bar{u^{\prime }v^{\prime }}}{\partial y} & \neq 0, \end{array}$$

(24) $$\begin{array}{ll} -\;\frac{\partial \bar{v^{\prime }u^{\prime }}}{\partial x}-\frac{\partial \bar{v^{\prime }v^{\prime }}}{\partial y} & \neq 0. \end{array}$$

If ${ℱ}_{s^{\prime }}$ includes a horizontal flux divergence component, the vertical integration of ${ℱ}_{s^{\prime }}$ is not appropriate.

After averaging variations in the frictional profile (presumably driven by convective and mesoscale flows that are associated with varying pressure gradient and advective tendencies at the circle scale, Figure 7), the mean influence of turbulence and convection on the frictional profile emerges. On average, the two assumptions on vanishing fluxes agree (Figure 10d) and the mean near‐surface momentum fluxes from JOANNE and the Saildrone agree (see the thick black dots in Figure 12a,b).

Organized mesoscale circulations are known to play an important role for convective momentum transport by deep convection (see, e.g., Badlan *et al*., 2017). Because of the use of periodic boundary conditions in traditional limited‐domain LES (<50 × 50 km${}^{2}$), the influence of mesoscale fluctuations on flux quantities has hardly been studied. Using a nested 100 × 100 km${}^{2}$ LES domain with open boundaries, Dixit *et al*. (2020) sampled the horizontal momentum flux and showed that horizontal circulations, which correspond to the air that flows laterally away from and towards buoyant updrafts to maintain mass continuity (and establish hydrostatic balance on a 100‐km scale domain), play an important role in generating momentum flux below 500 m towards the surface as well as near cloud tops. Whether organized shallow convection introduces mesoscale circulations and pressure gradients that substantially impact the wind, as suggested by our results, warrants further study.

## 7 SUMMARY AND CONCLUSIONS

EUREC${}^{4}$A/ATOMIC has made it possible to revisit the momentum budget of the trades studied in the 1950s (Riehl and Malkus, 1957) and 1970s (Holland and Rasmusson, 1973; Brümmer *et al*., 1974) and gain an observational perspective on the frictional layer and profiles of eddy momentum flux in fields of shallow convection with various forms of cloud organization (Schulz, 2021). We constructed the momentum budget from circular dropsonde arrays covering an area ∼220 km in diameter launched from the HALO aircraft with 70 flights over 13 days. The presence of multiple (∼six) subsequent circles allows a small but significant diurnal cycle in the wind throughout the lower troposphere to be observed. Wind speed reaches a minimum around 1600 LT as a result of weakening meridional winds during the night and early morning, followed by a weakening of zonal winds in the late morning. These changes go hand in hand with a diurnality in the pressure gradient force (Savazzi *et al*., 2021) and, although the diurnality is not fully understood, harmonious changes in large‐scale pressure gradient and convection coupled through the Hadley circulation appear to play a role (Dai and Deser, 1999; Ueyama and Deser, 2008; Savazzi *et al*., 2021).

Each circular array provides the local tendency of wind, mesoscale divergence, horizontal and vertical advection, pressure gradient, and Coriolis force. The tendencies are combined to calculate the flight‐mean residual, which is interpreted as an eddy momentum flux divergence, defined as a friction on the flow (Equation 21). The observed momentum budget is compared with that obtained from high‐resolution (9 km) day‐two forecasts of the IFS, which agree quantitatively below 1 km and in the shape of the dynamical and frictional forces at heights above 1 km, including the heights where forces change sign. Differences between the observations and the IFS are largely consistent with the zonal and meridional wind biases found during EUREC${}^{4}$A/ATOMIC (Savazzi *et al*., 2021).

The mean momentum budget is dominated by the pressure gradient and Coriolis force and the frictional force. Both observations and the IFS suggest that a frictional (Ekman) layer extends up to 1.5 km in the direction of the flow. At cloud base, the friction is still half its value near the surface, in line with studies that have suggested that shallow convective momentum transport establishes a frictional layer beyond the mixed layer, which justifies the need for large mechanical damping in the free troposphere in Matsuno–Gill type models (Carr and Bretherton, 2001; Lin *et al*., 2008). Wind turning is only 2.6° across the mixed layer, which implies that nonlocal momentum transport is efficient.

The wind speed tends to have a local maximum near 700 m to 1 km, which is approximately near cloud base. Between 1 and 1.5 km, the eddy flux divergence is countergradient and the observed wind shear is larger than the inferred shear in the geostrophic wind. This suggests that convection, by transporting low momentum from the mixed layer through cloud base and into the cloud layer, helps to sustain the local wind jet by diminishing the wind speed above the jet. This is in line with LES (Larson *et al*., 2019), which also showed that the momentum flux divergence carried just by cloudy updrafts is approximately zero at cloud base (e.g., moist convection does not slow down the jet itself) and only introduces friction above the jet (Dixit *et al*., 2020; Helfer *et al*., 2020).

The near‐surface eddy momentum flux derived by vertical integration of the mean residual is in agreement with mean 10‐m momentum fluxes measured by in situ platforms (τ∼0.1 N·m${}^{-2}$ at u=8.3 m·s${}^{-1}$). The in situ turbulence measurements reveal significant spatial variability in the momentum fluxes, with a non‐negligible contribution of mesoscale fluctuations (5–30 km), which would make assumptions used to derive the flux profile invalid and help explain why near‐surface fluxes derived from JOANNE on individual days do not agree with the in situ measures. Cross‐wind momentum fluxes ($\tau_{n}$) are up to 50% of the along‐wind fluxes on some flights and can contribute significantly to total momentum fluxes. The total momentum flux tends to be most variable halfway through the mixed layer, and can attain values near cloud tops that are just as large as in the mixed layer, in particular at times of more vigorous convection in February.

The contribution of along‐wind and cross‐wind eddy momentum fluxes to the total frictional force varies notably throughout EUREC${}^{4}$A/ATOMIC. During January, the derived frictional force appears to contribute to a slowing down and turning of the wind in line with Ekman pumping. As the trade‐wind layer deepens in early February and more vigorous shallow convection in the form of gravel (cold pools) and flowers is observed, the component of friction in the along‐wind direction decreases, and the cross‐wind component of friction becomes relatively more important and veers the wind, reducing Ekman pumping. The wind veering is interpreted as the action of convective and mesoscale flows that introduce momentum more efficiently from higher layers towards the surface and may compensate for small‐scale turbulent stresses. Additionally, a layer of eddy flux convergence is found that introduces an acceleration of easterly flow near cloud tops, which would deepen the layer of easterly wind. Overall, these findings are in line with simple theoretical models of the tropical atmosphere that assume deeper boundary layers are accompanied by weaker friction and stronger zonal flows (Wang and Li, 1993).

To the extent that the large‐scale circulation is driven by boundary‐layer wind convergence (Sobel and Neelin, 2006), convective flows can play an important role in setting the intertropical convergence zone and thus strength of the Hadley circulation. This makes parameterized (shallow) convective momentum transport an important candidate to take into account when addressing double ITCZ problems in climate models. Ongoing work employs large‐eddy and mesoscale weather model simulations based on EUREC${}^{4}$A/ATOMIC to study how turbulence, convection, and mesoscale flows associated with different cloud patterns determine the observed momentum flux divergence.

## AUTHOR CONTRIBUTIONS

**L. Nuijens:** conceptualization; formal analysis; funding acquisition; investigation; methodology; visualization; writing – original draft; writing – review and editing. **A. Savazzi:** formal analysis; investigation; methodology; visualization. **G. de Boer:** conceptualization; data curation; formal analysis; funding acquisition; investigation; project administration; resources; visualization; writing – original draft; writing – review and editing. **P‐E. Brilouet:** data curation; formal analysis; visualization; writing – original draft; writing – review and editing. **G. George:** data curation; formal analysis; investigation; methodology; software; validation; writing – original draft; writing – review and editing. **M. Lothon:** data curation; formal analysis; supervision; writing – review and editing. **D. Zhang:** formal analysis; funding acquisition; investigation; writing – original draft; writing – review and editing.

## Supporting information


**Appendix S1** Supporting Information
