# Supplementary material for: The frictional layer in the observed momentum budget of the trades
Source: Q J R Meteorol Soc. 2022 Oct 1;148(748):3343–65. doi: 10.1002/qj.4364 (PMC9828681; doi:10.1002/qj.4364)
Supplement: Supplementary file 1 — Appendix S1 Supporting Information [file QJ-148-3343-s001.pdf]

Supporting Information for: The frictional layer in the observed momentum budget of the trades

L. Nuijens, A. Savazzi, G. de Boer, P-E. Brilouet,G. George, M. Lothon, D. Zhang

July 22, 2022

1 Large-scale pressure gradients and thermal wind

The eddy momentum flux divergence derived from the budget in section 4 of the accompanying paper is a strong function of the pressure gradients measured across the circle. The pressure gradient and geostrophic wind can also be derived from ERA5 reanalysis over the circle area and over a larger area over the North Atlantic. Averaged over all HALO flight days, the north-south pressure gradient that drives the zonal geostrophic wind u_g is shown in Figure 1d. The three lines correspond to the pressure gradients from JOANNE (dotted), ERA5 over the circle area (solid) and ERA5 over a 1000^2 km^2 area encompassing the circle (dashed line). The three estimates agree very well below 1 km and above 4 km, but differ by a few per day in the cloud layer, whereby ERA5 on the circle scale and JOANNE best agree on the profile of the pressure gradient the local minimum in the pressure gradient at 2.5 km (near the mean trade-inversion). This feature is thought to be associated with mesoscale variations in the pressure field driven by convective heating and radiative cooling. It is just above the maximum in mean thermal wind shear determined over the 1000^2 km^2 area (Figure 2, which is approximately the shear in the zonal geostrophic wind ($\partial_z u_g$, not shown).

The east-west pressure gradient that drives the meridional geostrophic wind v_g (Figure 1a) reveals a positive pressure gradient and a northward geostrophic wind in the circle in both JOANNE and ERA5, while the large-scale pressure gradients is always directed southward. As in the north-south direction there are differences on the order of a few per day within the cloud layer (note that the scale of the x-axis is different from Figure 1d).

The agreement in the profile of the pressure gradient is reassuring. From January to mid-February the trade-winds strengthened and became more northeasterly (than easterly) as pressure gradients increased, which can be seen in the large-scale (ERA5) as well as the circle-measurements (JOANNE). Differences between JOANNE and ERA5 seem to highlight the presence of mesoscale variability in pressure and wind related to local processes, which may not be present in the model and help explain lower tropospheric wind biases in ERA5 compared to the observations (Savazzi et al, 2021). This variability cancels out in the mean to a large degree, but is pronounced

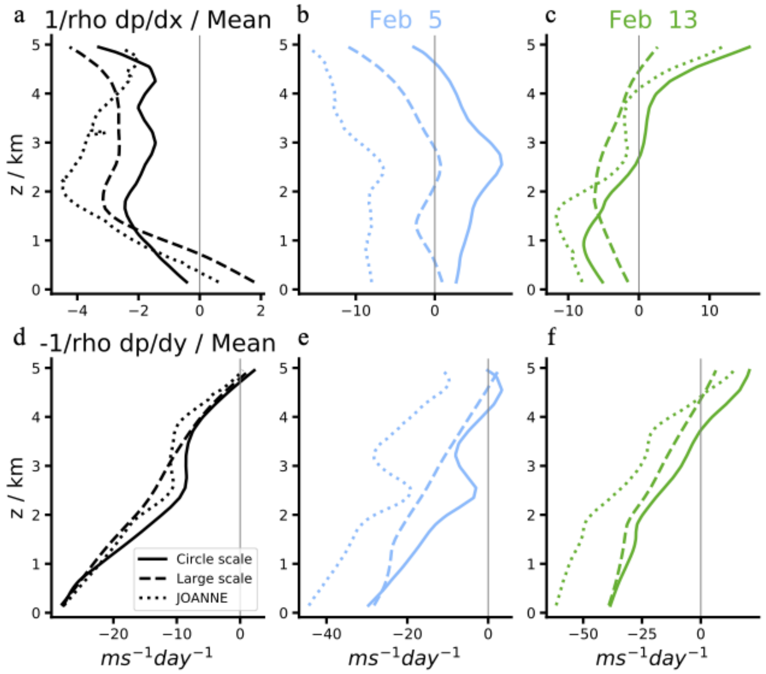

Figure 1: The pressure gradient in the x and y direction, corresponding to v_g (top) and u_g (bottom) as derived as means over the circle from both JOANNE and ERA5, and from ERA5 over a large-scale area 1000 x 1000 km^2. Differences in the mean gradients are small near the surface and above the trade-inversion in the y direction, but larger in the cloud layer.

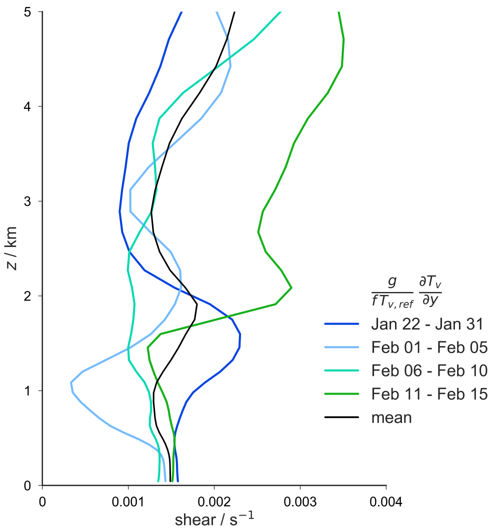

Figure 2: Profiles of geostrophic wind shear or thermal wind $(-\frac{g}{fT}\partial_y T)$ determined from ERA5 over a 1000 x 1000 km^2 area shows a local maximum in large-scale wind shear between 1 and 2 km, with considerable variations among periods.

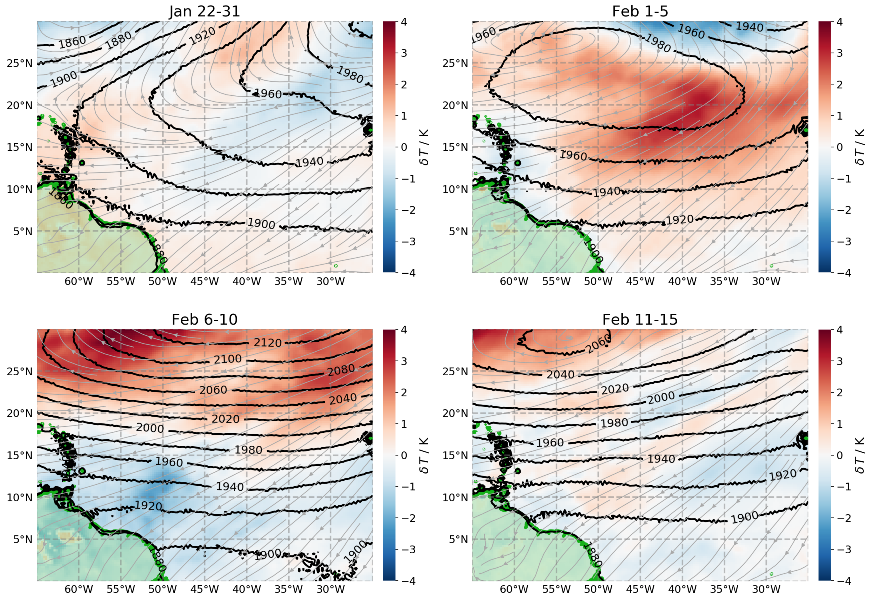

Figure 3: Temperature at 2 km as an anomaly with respect to the period, low-level wind vectors and 825 hPa geopotential heights (in m) over the North-Atlantic indicate the presence of an anomalously warm central and northern Atlantic during the first ten days of February and a westward displacement of the subtropical high.

when individual flight days are considered (in blue and green). On Feb 5th, JOANNE and ERA5 over the circle do agree on a more pronounced (than in the mean) local minimum near 2.5 km. On this day gravel and flowers dominated east of Barbados and it is not unlikely that mesoscale dynamics explain the differences.

Between Feb 5 and 13, extratropical anomalies in pressure and resultant cold or warm air advection played a role in reducing the lower-tropospheric thermal wind (and thus shear) between Feb 6 - 10 (aquamarine line in Figure 2). Maps with ERA5 temperature anomalies at 2 km and geopotential height anomalies at 825 hPa (taken with respect to a monthly mean centered on) are shown in Figure 3. From the end of January into February, strong mid-level warm air advection from the east reduced the thermal contrast between the upstream and downstream trades. With increasing wind speed and still strong cold air advection near the surface, the cloud field transitioned from sugar to gravel and flower patterns in early February. From February 6 to 10 a very strong subtropical high at mid-levels displaced to the west drove warm air advection further westward and a reduction in zonal wind shear. The area upstream of Barbados experienced gravel and flowers and deeper clouds embedded in fish. By February 15, strong northeasterly flow brought cold air masses with a diverse cloud field including flower-like structures that transitioned towards fish-like structures with larger precipitating cells and cold pools.

2 Evolution of budget components

The evolution of the momentum balance for individual days is illustrated with a time series in Figure 4, which shows the forcing terms as averages over the surface layer (0 - 200m) for each flight day. This includes the tendency in $\bar{u}_s$ (a), the horizontal and vertical advection combined (b), the pressure gradient and Coriolis force (c-d), the total residual $\mathcal{F}$ (e) and its components $\mathcal{F}_s$,$\mathcal{F}_n$ (f - g). It also shows the vertical velocity w averaged over the lower 1.5 km of the atmosphere and the inversion height z_{inv}, determined as the level at which the Brunt-Väisälä frequency squared maximizes below 3000 m George2021c. The pressure gradient, Coriolis and advection terms are also shown for the P3 flights to illustrate that the forcing terms for individual circles can be large and variable, reflecting the mesoscale flow field.

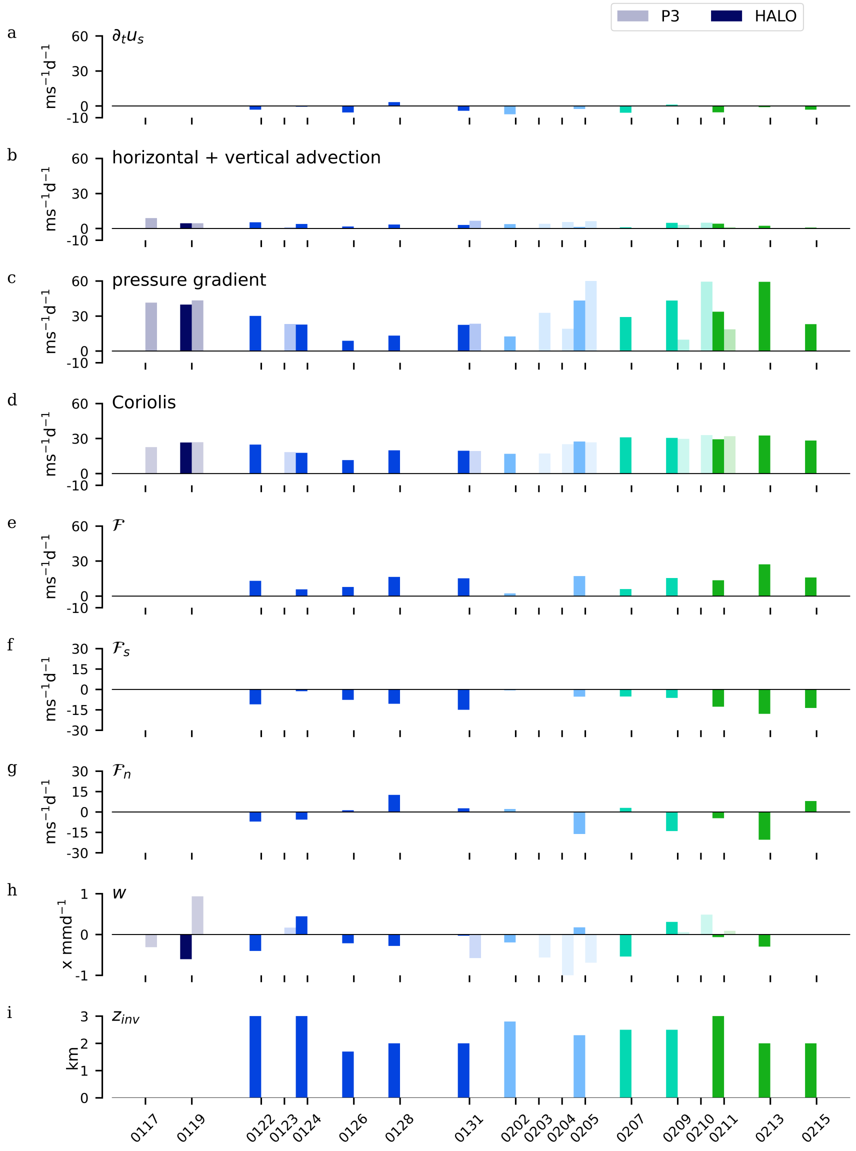

Figure 4: The time series illustrates variability in the forcing terms, here shown as averages over the surface layer (0 - 200m) for each flight day. Terms included are the tendency in $\bar{u}_s$ (a), the horizontal and vertical advection combined (b), the pressure gradient and Coriolis force (c-d), the total residual $\mathcal{F}$ (e) and its components $\mathcal{F}_s$,$\mathcal{F}_n$ (f - g). Also shown are the vertical velocity w averaged over the lower 1.5 km of the atmosphere and the inversion height z_{inv}, determined as the level at which the Brunt-Väisälä frequency squared maximizes below 3000 m George2021c. The pressure gradient and inferred frictional force constitute a first-order balance on most days, because total horizontal and vertical advection are less important. Along with weak or reversed pressure gradients in the along-wind direction, the inferred frictional force is smaller in the first week(s) of February. All tendencies are first aligned with the wind at every height level.
